# Self-supervised deep learning model for COVID-19 lung CT image segmentation highlighting putative causal relationship among age, underlying disease and COVID-19

**DOI:** 10.1186/s12967-021-02992-2

**Published:** 2021-07-26

**Authors:** Daryl L. X. Fung, Qian Liu, Judah Zammit, Carson Kai-Sang Leung, Pingzhao Hu

**Affiliations:** 1 Department of Computer Science, University of Manitoba, Winnipeg, MB R3T 2N2 Canada; 2 Department of Biochemistry and Medical Genetics, University of Manitoba, 745 Bannatyne Avenue, Winnipeg, MB R3E 0J9 Canada; 3 CancerCare Manitoba Research Institute, CancerCare Manitoba, Winnipeg, MB R3E 0W3 Canada

**Keywords:** COVID-19, Self-supervised learning, Lung CT images, Image segmentation, Mediation analysis

## Abstract

**Background:**

Coronavirus disease 2019 (COVID-19) is very contagious. Cases appear faster than the available Polymerase Chain Reaction test kits in many countries. Recently, lung computerized tomography (CT) has been used as an auxiliary COVID-19 testing approach. Automatic analysis of the lung CT images is needed to increase the diagnostic efficiency and release the human participant. Deep learning is successful in automatically solving computer vision problems. Thus, it can be introduced to the automatic and rapid COVID-19 CT diagnosis. Many advanced deep learning-based computer vison techniques were developed to increase the model performance but have not been introduced to medical image analysis.

**Methods:**

In this study, we propose a self-supervised two-stage deep learning model to segment COVID-19 lesions (ground-glass opacity and consolidation) from chest CT images to support rapid COVID-19 diagnosis. The proposed deep learning model integrates several advanced computer vision techniques such as generative adversarial image inpainting, focal loss, and lookahead optimizer. Two real-life datasets were used to evaluate the model’s performance compared to the previous related works. To explore the clinical and biological mechanism of the predicted lesion segments, we extract some engineered features from the predicted lung lesions. We evaluate their mediation effects on the relationship of age with COVID-19 severity, as well as the relationship of underlying diseases with COVID-19 severity using statistic mediation analysis.

**Results:**

The best overall F1 score is observed in the proposed self-supervised two-stage segmentation model (0.63) compared to the two related baseline models (0.55, 0.49). We also identified several CT image phenotypes that mediate the potential causal relationship between underlying diseases with COVID-19 severity as well as the potential causal relationship between age with COVID-19 severity.

**Conclusions:**

This work contributes a promising COVID-19 lung CT image segmentation model and provides predicted lesion segments with potential clinical interpretability. The model could automatically segment the COVID-19 lesions from the raw CT images with higher accuracy than related works. The features of these lesions are associated with COVID-19 severity through mediating the known causal of the COVID-19 severity (age and underlying diseases).

**Supplementary Information:**

The online version contains supplementary material available at 10.1186/s12967-021-02992-2.

## Background

Coronavirus disease 2019 (COVID-19) is a newly identified infectious disease, which was first reported in December 2019 [1]. According to an interactive COVID-19 dashboard created by Johns Hopkins University, COVID-19 has spread to more than 190 counties and caused 3,957,898 global deaths out of more than 182 million diagnosed cases by July 2nd, 2021 [2]. Several interventions have been applied worldwide to control the COVID-19 pandemic, such as case isolation, close contact quarantine, population lockdown, face covering, sanitization, and vaccination. Although these preventative measures have successfully reduced the number of deaths and confirmed cases, we will likely experience more waves of COVID-19 as restrictions are loosened and the new variants appear [3].

Recently, many efforts have been made to develop artificial intelligence (AI) models to support medical imaging-based COVID-19 rapid diagnosis [4]. Compared to Polymerase Chain Reaction (PCR) which is the current gold standard COVID-19 diagnosis test, medical imaging such as computerized tomography (CT) scans of the lungs does not waste consumables. Therefore, CT imaging-based COVID-19 diagnosis is more efficient as it would not be limited by the delay of available testing kits, especially when AI is introduced to release the need for human involvement in image reading [5]. However, current AI COVID-19 diagnostic models based on medical imaging generally lack transparency and clinical interpretability [6]. The complexity of AI models and their low reproducibility have weakened their applications in clinical practice [7]. Hence, it is critical to develop AI-based COVID-19 diagnosis models with clinical interpretability. Age, underlying diseases, and sometimes gender are observed to be related to the risk of COVID-19 [8, 9]. Lung CT image is a good predictor of COVID-19 status. This is likely due to its associations with age, gender, and underlying diseases [10, 11]. If this is the case, mediation analysis [12] between the age, gender, underlying disease and the risk of COVID-19 through lung CT image phenotypes can potentially be used to reason on the model predictions both biologically and statistically. This may have the potential to improve the cost-effectiveness, diagnosis efficacy, and clinical utility of AI-based COVID-19 CT imaging diagnosis [6].

There are several related works that used self-supervised learning approaches for predicting if the CT lung images is COVID-19 positive or COVID-19 negative. Chen et al. [13] proposed to use contrastive self-supervised learning with 3 major components—data augmentation, representation learning, and few-shot classification. The data augmentation that they used involved cropping two parts of the CT lung images, one part undergone random cropping followed by random flipping, the other part undergone random cropping followed by colour distortion. Then, representation learning was trained to improve on the similarity score where cropped images from the same CT lung image achieved higher similarity score and cropped images from different CT lung images achieved a lower similarity score. After training representation learning on the model, the pre-trained model was used to encode the query image and the support set of CT lung images. The encoded features were passed into prototypical networks to conduct the few-shot classification. The limitation of the work in this approach is that support set images are required for the classification. It can also be hard for the classification to get a good performance if the encoded features of the query image are very different from the support set images. Li et al. [14] used self-supervised dual-track learning to rank. Since there are more available COVID-19 negative samples than COVID-19 positive samples, their method selected a subset of the negative samples to train on the network so that a more balanced data was trained. The way the subset of the negative samples was selected is that they generated two soft labels (“difficulty” and “diversity”) for the negative samples by computing the earth mover’s distance between the COVID-19 negative samples and the COVID-19 positive samples and selected the soft labels generated accordingly.

As it has been more than one year since the occurrence of COVID-19, many lung CT image datasets are now available online. Though effective, deep learning requires data sets with a large sample size to achieve better performance [15]. However, only few COVID-19 CT image dataset has segmentation label information. Therefore, it is important to effectively utilize them. Voulodimos et al. proposed two deep learning models to do COVID-19 infected area segmentation from CT image patches [16, 17]. These patches acted as augmentations of the raw images. Ma et al. further proposed the data-efficient learning which involves few-shot learning, domain generalization for COVID-19 segmentation with limited training data [18]. Transfer learning is also widely used to complement the sample size issue in COVID-19 infection detection and segmentation from medical images [19–21]. However, transfer learning usually involves models pretrained on non-medical images, which may not perform well in the medical image scenario. Incorporating unlabeled data into model training strategies is also an approach to improve the prediction performance when the labeled data have limited size [22]. Yao et al. even proposed a label-free deep learning-based segmentation model which took advantage of unsupervised anomaly detection techniques [23]. However, their model only outperformed other unsupervised approaches and maybe not comparable with supervised methods. Self-supervised learning is another way to involve unlabeled data. It aims at creating tasks to generate auto-achievable labels without additional human annotations [24]. In the context of self-supervision, image inpainting [25] refers to the creation of a task for the model to generate the content of missing or damaged regions based on the surrounding information [26–28]. The images are damaged on purpose by making some missing regions. Then the model is trained to recover the damaged images to their raw versions. Image inpainting was reported to have excellent pre-training ability for convolutional neural network (CNN) based image segmentation, because it can improve network feature learning [26, 29]. By controlling the complexity of missing regions in the images, we can manage the difficulty of the inpainting task. However, it is hard to create proper missing regions for network to learn, because the missing regions can either be too complex for the network to start learning or too simple to be able to learn good representations [30]. A coach network with generative adversarial mechanism [31] can be used to create the missing region masks with proper complexity. The created mask can initially be simple. Once the network can predict the inpainting of the CT images with good performance, the coach network increases the complexity of the masking to reduce the performance of the network, similar to how the generative adversarial network (GAN) [31] works.

Automatic lung CT image lesion segmentation is not easy due to its variation [32]. To distinguish different kinds of lesions is even harder. To exhaust the information in COVID-19 lung CT images and help us to understand the disease thoroughly, it is important to segment and understand COVID-19 lung CT lesions at a pixel level, including ground-glass opacity (GGO) and consolidation. The recently developed COVID-19 Lung Infection Segmentation Network (InfNet) [22] uses a two-stage strategy to solve the multi segmentation problem. That is, the overall lesion is first segmented, and then passed to the second stage for further distinguishing into the GGO or consolidation lesion [22].

In diagnostic radiology, consolidation could be either pus, edema, blood, or a tumor replacing the airspace in the lung, while GGO is either the filling of pus, edema, hemorrhage, inflammation, or tumor cells in the alveolar space [33, 34. These two lung CT patterns often present together, but GGO is more commonly observed in COVID-19 lung CT images than consolidation [35]. This is the case in the segmented lung CT image dataset we found online [36]. This imbalanced label problem might overwhelm the default binary cross entropy loss function in training the binary classifier. When distinguishing the GGO and consolidation from overall lesion segment, the negative samples— which are the consolidations—are easier to classify than the positive samples (GGO) because the large number of negative samples contribute to the majority of the loss and have a huge influence on the gradient. Focal loss is an improved loss function that could reduce the weight of easy samples and focus more on samples in minority class. It can improve the performance of the classification network when the dataset has class imbalance [37].

In addition to choosing the loss function wisely, another important technical point for training the network is to configure the most advanced iterative method to optimize the loss function. Currently, many successful networks are trained using the stochastic gradient descent (SGD) algorithm [38], and its variants. To improve SGD, and other optimizers, a novel algorithm called Lookahead was proposed [39. It uses two nested loops to update two sets of network weights. The fast weights of the network are trained several times in a small inner loop using an optimizer such as SGD, then the direction of the gradient is used to update the slow weights using the outer loop [39]. It is almost guaranteed to achieve fast convergence with minimal computational overhead [39].

**Fig. 1:**
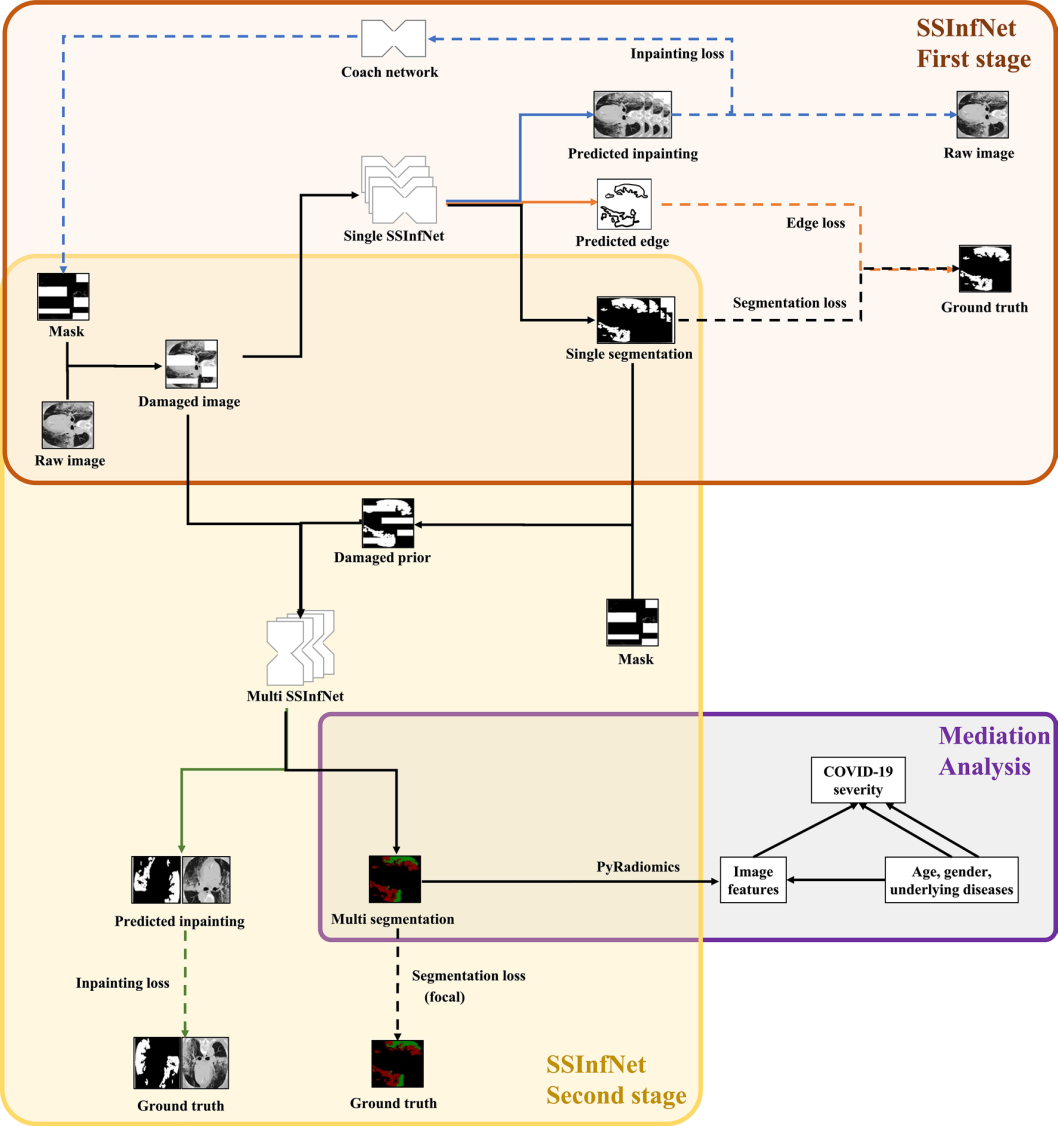
Overview of the proposed self-supervised COVID-19 lung infection segmentation (SSInfNet) model and statistic causal mediation analysis of the predicted segments. The black path shows the main workflow of the proposed two-stage SSInfNet model and the follow-up statistical mediation analysis. The first stage is a single SSInfNet which takes the damaged CT image as input, and outputs the reconstructed image (blue path), the edges of overall lesion segment (orange path), and the single segment itself. The inpainting loss and edge loss are intended to increase the complexity of the single SSInfNet to improve its segmentation ability. The coach network (presented in the blue path) forms a generative adversarial mechanism with single SSInfNet to further improve the later model’s performance. Continuing to proceed along the black path, the raw CT image and the predicted overall lesion segment (as prior) are used as input for the multi SSInfNet to further divide the overall lesion segments into ground-glass opacity and consolidation segments. Image inpainting is also involved in this stage (green path). For the multi segmentation, we use the focal technique as its loss function and lookahead optimizer as its training strategy. At the end, the predicted multi segments are used to extract several images features with Python’s PyRadiomics [41] package. The image features act as mediators in the mediation analysis model between the independent variables (age, gender, and underlying diseases) and the dependent variable (COVID-19 severity)

In the current study, we propose an advanced deep learning model called self-supervised InfNet (SSInfNet) which uses InfNet as a backbone, and integrates generative adversarial image inpainting, focal loss, and lookahead optimizer techniques (Fig. 1) to improve lung lesion segmentation performance compared to benchmark models. Furthermore, the clinical mechanisms of the predicted multi lung lesion segments (GGO segment and consolidation segment) on COVID-19 are evaluated in this study using statistic mediation analysis. The identified mediation effects could significantly increase the interpretability of the network and support certain image features as potential diagnostic image biomarkers for COVID-19.

## Methods

### Data split

**Fig. 2:**
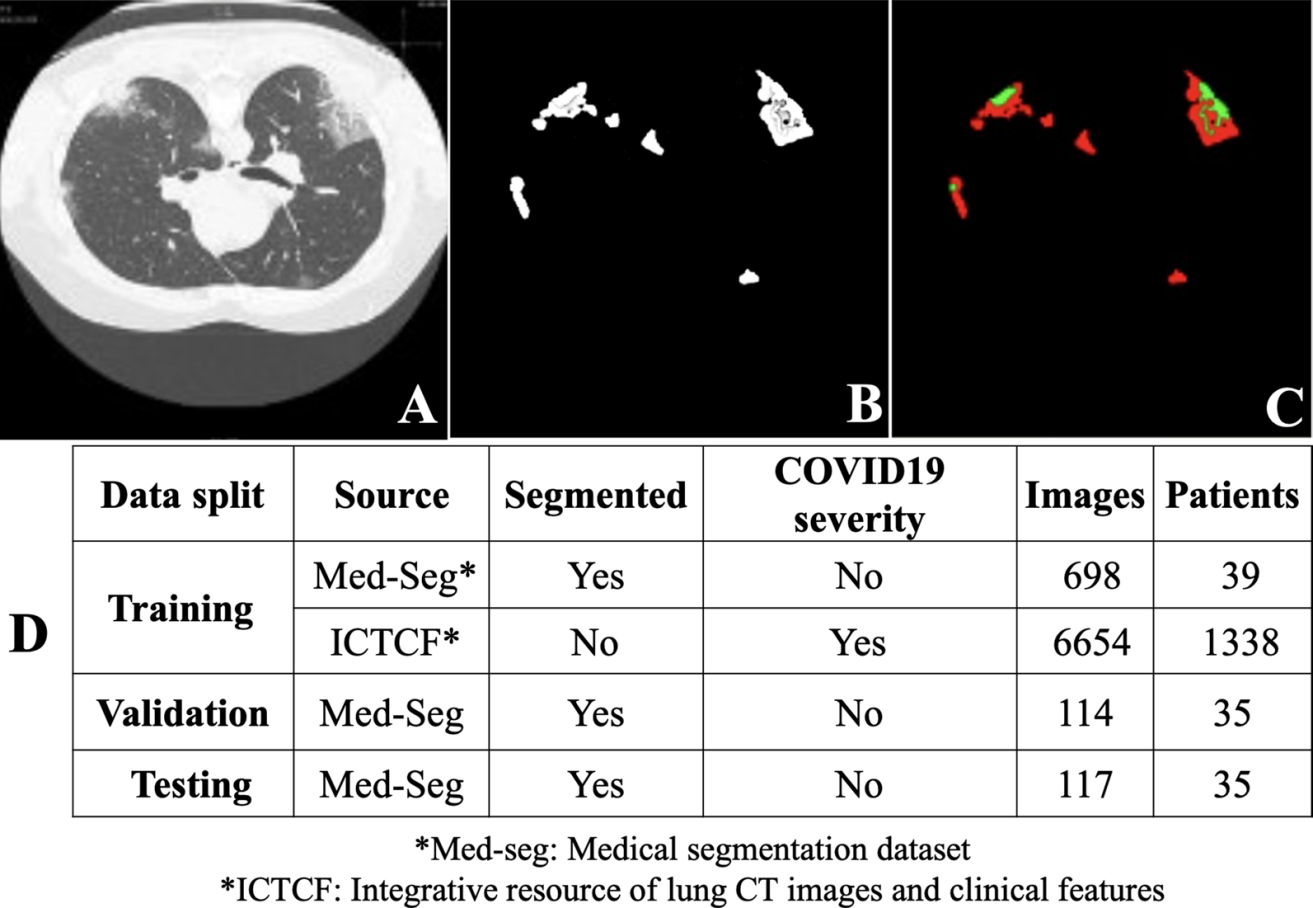
Segment visualization and data split. **A** Examples of raw lung CT images in both Med-seg dataset and ICTCF dataset. Images are all in the axial view which looks down through the body. **B** The overall lesion segment. This is the label for the proposed single self-supervised COVID-19 network (SSInfNet) model for lung infection segmentation, and it exists only in Med-seg dataset. **C** The ground-glass opacity segment (red) and consolidation segment (green). This is the label for multi SSInfNet and it is also only available for the Med-seg dataset. **D** The table shows the data utilization in the development of the proposed SSInfNet models. As ICTCF does not contain segment labels, it was used only for the self-supervised image inpainting in the training stage. The Med-seg image data was split into training, validation, and testing sets, approximately under the ratio of 6:1:1. After the model was well developed, it was applied to the ICTCF dataset for further statistic mediation analysis because only ICTCF contains COVID-19 clinical severity information, which means Med-seg data was not used in the mediation analysis

Two COVID-19 datasets were involved in this study. One is the Integrative Resource of Lung CT Images and Clinical Features (ICTCF) [40] which contains the clinical severity for each patient. There are 6654 lung CT images from 1338 patients with their clinical severity in ICTCF. The other is Med-Seg (medical segmentation) COVID-19 dataset [36] which contains 932 CT lung images with the ground truth labels of their GGO and consolidation segments. The segments and data splits are shown in Fig. 2. We split the dataset into training set and test set. To prevent data leakage, we split the dataset based on the patients rather than the CT lung images.

### Supervised InfNet

The supervised InfNet (SInfNet) is a recently developed CNN model for COVID-19 lung CT segmentation [22], which was used as both our backbone and one of the baseline models. We did not change the overall structure and default hyperparameters of the original SInfNet model (Additional file 1: Figure S1). A complete SInfNet consists of two parts: a single SInfNet (Additional file 1: Figure 2A) and a multi SInfNet (Additional file 1: Figure 3A). The single SInfNet only predicts the infected region without classifying them more specifically. The input of the single SInfNet is a raw CT lung image and the output includes the edge contour of the overall lesion regions and four overall lesion region segmentations with different sizes as shown in Additional file 1: Figure S1. A CT lung image is first passed into the initial convolutional layers of the single InfNet to extract image features. Then, the features generated from the convolutional layer are fed into the partial decoder module, reverse attention module, and the edge detection module. The edge detection module is meant to help the network with the detection of the boundaries of the segmentation. The reverse attention and the partial decoder generate the segmentation of the infection regions of the CT lung images.

The prediction from the single SInfNet represents the overall infected regions and acts as a prior to be fed, concatenated with the original CT images, into the multi SInfNet. The multi SInfNet is used to predict multiple labeled segmentations. The segmentations include the predicted background, GGO, and consolidation.

### Self-supervised InfNet

The self-supervised InfNet (SSInfNet) is our proposed COVID-19 segmentation CNN model, which, like the SInfNet, includes two parts, a single SSInfNet (Additional file 1: Figure S2B) and a multi SSInfNet (Additional file 1: Figure S3B). It is obtained by integrating generative adversarial image inpainting, focal loss, and Lookahead optimizer to SInfNet. The original SInfNet model generates 5 different predictions: an edge segmentation prediction and the 4 segmentations of the infected regions. To utilize the ability of a self-supervised method for the SInfNet’s segmentation, we generate masks fed into the SInfNet model. The last convolution layer that outputs the prediction is not used for the self-supervised case. However, the last convolutional layer is replaced with a different convolutional layer to reconstruct the image and the edge appropriately. Everything else is kept the same as the SInfNet architecture (Additional file 1: Figure S2A). This process allows the network learns meaningful representations of the CT images. We can use these meaningful representations to segment the infected regions of CT lung images. After learning the self-supervised features for InfNet, the training continues as normal, similar to the SInfNet algorithm. The training starts with the weights trained using the self-supervised inpainting method. The last layer is changed to its original layer instead of the replaced convolutional layer.

By learning features from image inpainting, the model can learn features that are closer related to image segmentation. As creating masks can be a complex task for the network to learn to inpaint, the mask can either be too complex for the network to start learning or too simple to be able to learn good representations. We use a coach network that increases the complexity of the masking of the CT images throughout the training of the network. The mask created is initially simple, once the network can predict the inpainting of the CT images with good performance, the coach increases the complexity of the masking to reduce the performance of the network. The loss for the coach network is constructed from the loss of the image inpainting from the SSInfNet. The coach network and the SSInfNet both works together as a MinMax algorithm. The SSInfNet tries to minimize the loss to generate better image inpainting while the coach network tries to increase the loss of the image inpainting through generating more complex masks. In the beginning, the masks generated by the coach network are quite simple. Through the training of the coach network, as the SSInfNet gets better at predicting image inpainting, the coach network starts to generate more complex masks. The loss function for the coach network is:

1 $$L_{coach} (x) = 1 - L_{rec} (x \odot M)$$

where.

- *M* is the mask created by the coach network
- *x* is the CT lung image
- *L*_*coach*_ is the loss for the coach network
- *L*_*rec*_ is the loss for the reconstruction loss

A constraint is applied to this loss function because the coach network would just create a mask that masks all regions. After all, no context information would be present for the network to learn and a maximum loss is achieved. The constraint is:

2 $$\hat{B}(x) = B(x) - SORT(B(x))^{k|B(x)}$$

3 $$M = C(x) = \sigma (\alpha \hat{B}(x))$$

The backbone, *B*, of the coach network has a similar network architecture as the InfNet models. SORT*(B(x))* sorts the features in descending order over the activation map. *k* represents the *k*^*th*^ elements in the sorted list and *k* helps to control the fraction of the image to be erased. The regions that have scores smaller than the *k*^*th*^ element are erased from the images. If *k* is 0.75, then 0.75 percent of the image is not erased. The score is scaled into a range of [0,1] using a sigmoid, σ, activation function. *C(x)* is the coach network that is fed with the CT lung images. The illustration of the coach network can be seen in Fig. 1.

After the self-supervision training is finished, the single SSInfNet is reused to train normally, using the segmentation of the CT lung images. Likewise, the multi SSInfNet network reuses the weights that are trained during the self-supervised multi SSInfNet to train normally, using the multi segmentations of the CT lung images.

The proposed single SSInfNet architecture can be seen in Fig. 1 and Additional file 1: Figure S2B. Additional file 1: Figure S2A shows the original single SInfNet architecture. The difference is that the last layer for each output prediction is replaced with a different linear activation layer. The linear activation layer recreates the original image that is covered by the masks. The proposed multi SSInfNet architecture is shown in Fig. 1 and Additional file 1: Figure S3B. The changes in the architecture for the multi SSInfNet are similar to the single SSInfNet where the last convolutional layer is replaced with a different linear activation layer to output the inpainting of the original image.

A loss is calculated for each of the outputs of the single SInfNet model. The first loss function is the edge loss, *L*_*edge*_, which guides the model in representing better segmentation boundaries. The other loss function is the segmentation loss, *L*_*seg*_. The segmentation loss combines both the loss of Intersection over Union (IoU) and the binary cross entropy loss (*LBCE*). The segmentation loss equation for the single SInfNet is as follow:

4 $$L_{seg} = L_{IoU} + \lambda L_{BCE}$$

λ is a hyperparameter that controls how much weight we want to put on the binary cross entropy loss. The segmentation loss is adapted to all *S*_*i*_ predicted output where *S*_*i*_ are created from *f*_*i*_ such that *i* = 3, 4, 5. As low-level features use more computational resources due to larger spatial resolutions but achieves lesser performance. We use the features in the higher level (*i* = 3, 4, 5) instead. The total loss function for the single SInfNet model is then:

5 $$L_{total} = L_{seg} (G_{t} ,S_{g} ) + L_{edge} + \sum\limits_{i = 3}^{5} {L_{seg} (G_{t} ,S_{i} )}$$

The summation of the segmentation loss functions is calculated from the output of the parallel partial decoder and the three convolutional layers (*i﻿* = 3, 4, 5)). *G*_*t*_ refers to the ground truth labels. *S*_*g*_ is the output from the parallel partial decoder to match with the ground truth label. *S*_*i*_ is the different sizes of the segmentation of infected regions output by the InfNet. The different sizes of the segmentation of infected regions outputted by the SSInfNet are resized to the same shape as the ground truth segmentation image.

As for the multi SSInfNet, we use the default model and hyperparameters from the multi SInfNet. However, we train the multi SSInfNet without using any unlabeled images during self-supervision because the multi SSInfNet requires the prior (infected region) as input. The CT lung images and prior (infected region) for the CT lung images are concatenated together before being fed into the multi SSInfNet. The prior is generated from the single SInfNet. The multi SSInfNet labels the prior with background, ground-glass opacities, and consolidations. The loss function for the multi SSInfNet is as follow:

6 $$L_{bce} = \frac{1}{N}\sum\limits_{i = 1}^{N} {y_{i} \cdot \log (\hat{y}_{i} )} + (1 - y_{i} ) \cdot \log (1 - \hat{y}_{i} )$$

Where,

- *y*_*i*_ is the ground truth value for the segmentation—background, ground-glass opacities, or consolidation
- $\widehat{{y}_{i}}$ is the network's predicted value for the segmentation
- *N* is the total number of the current training batch of data samples

The loss function for the multi SInfNet uses the binary cross-entropy loss between the predicted and the ground truth segmentation. In order to improve the performance of the model and to aid in its generalization, we chose to use self-supervised learning to learn good representations of the CT lung images.

Additionally, we use the focal loss instead of the binary cross-entropy loss function for the Multi SSInfNet model to provide more weight on the smaller data label samples (consolidation). The focal loss function is:

7 $$FL(p_{t} ) = - \alpha_{t} (1 - p_{t} )^{\gamma } \log (p_{t} )$$

where.

- *FL* is the focal loss
- *p*_*t*_ is the Multi SSInfNet's predicted output
- α_*t*_ is a hyperparameter that controls the weight of positive and negative samples
- γ is the term that controls the rate of the downweighed examples

We also wrap the Lookahead optimizer around the SGD optimizer with *k* = 5 and alpha = 0.5. *k* is the number of inner-loops the SGD will optimize before the Lookahead optimizer starts optimizing. In our case, after the SGD optimizes the network weights for 5 iterations, the Lookahead optimizer will optimize using alpha multiplied by the difference between the network weights after the 5 iterations of SGD optimizer and the network weights before the 5 iterations of the SGD optimizer. The alpha is used to control the intensity of the difference. The pseudo code for our single/multi SSInfNet can be found in Additional file 1: Algorithm 1.

### Experimental settings

For the Single SInfNet, we train the network for 500 epochs. We use Adam as the optimizer with a learning rate of 0.0001. For the Multi SInfNet, we train the network for 500 epochs. We use SGD as the optimizer. The momentum is set as 0.7 and the learning rate is set as 0.01. As for the Multi SSInfNet with added focal loss and lookahead optimizer, we train the network for 500 epochs, use lookahead optimizer with *k* = 5 and alpha = 0.5, and wrap the Lookahead optimizer around the SGD optimizer where the momentum is set as 0.7 and the learning rate is set as 0.01.

For the self-supervised version of both the Single SInfNet and Multi SInfNet, the self-supervised image inpainting is first trained. Then the weights from the trained networks, except for the last layer, are transferred and be used to train on the segmentation of the CT lung images. During the self-supervised image inpainting stage, we train the network for 2000 epochs. The network is trained for the first 200 epochs before we train the coach network for 200 epochs which increases the complexity of the masks generated. After that, we alternate in between training the self-supervised image inpainting for 100 epochs and the coach network for 100 epochs for 1800 epochs in total. For every alternating between the training of the self- supervised image inpainting and the coach network, we set the learning rate to 0.1 at the start of the epoch, 0.01 at the 40th epoch, 0.001 at the 80th epoch, and 0.0001 at the 90th epoch to speed up convergence. We use SGD as the optimizer for the self-supervised image inpainting, set the momentum to 0.9 and the weight decay to 0.0005. As for the optimizer for the coach network, we use the Adam optimizer with a learning rate of 0.00001.

We compare our self-supervised method against some supervised models trained on the COVID-19 datasets. We train and follow the same network structure, but change from supervised learning to self-supervised learning, and compare the performance between the supervised and the self-supervised approaches. We want to determine if self-supervised learning is a useful way to help the SInfNet improve its performance in segmenting the ground-glass opacities or consolidation around the infected region of the CT lung images.

### Performance evaluation metrics

Five metrics are used to measure the models’ performance: F1, intersection over union (IoU), Recall, and Precision and the area under the curve (AUC) of a receiver operating characteristic (ROC):

The F1-Score is also called the Dice Coefficient. It is used to measure the overlap between the ground-truth infected region and the predicted infected region. The F1-Score equation is defined as:

8 $$F1 = \frac{{2*\left| {T \cap P} \right|}}{\left| T \right| + \left| P \right|}$$

where T is the ground truth infected region and P is the predicted infected region.

The Intersection over Union (IoU) is a different method to measure the overlap between the ground truth infected region and the predicted infected region. The IoU equation is defined as:

9 $$IoU = \frac{T \cap P}{{T \cup P}}$$

where T is the ground truth infected region and P is the predicted infected region.

The Recall is used to measure how much of the ground truth infected region is present in the predicted infected region. The equation is as follow:

10 $${\text{Re}} call = \frac{T \cap P}{T}$$

where T is the ground truth infected region and P is the predicted infected region.

The Precision is used to measure how much of the predicted infected region is present in the ground truth infected region. The equation is as follow:

11 $$\Pr ecision = \frac{T \cap P}{P}$$

where T is the ground truth infected region and P is the predicted infected region.

For each of above performance metrics, we first perform the calculation within each test sample, separately. We compute the mean and the error interval of the estimated mean for each of the metrics in the entire test set. The mean is defined as:

12 $$mean = \frac{{\sum\nolimits_{i = 1}^{N} {Metric(\hat{y}_{i} ,y_{i} )} }}{N}$$

where Metric refers to F1, IoU, Recall, Precision or AUC. N refers to the number of test samples. The error is defined as:

13 $$error = SE \times 1.96$$

where SE is the standard error of the test samples for the given metric. The error interval of the estimated mean is defined as − *error* and + *error*.

### Generation of image phenotypes

The well-trained multi SSInfNet outputs three kinds of image-level segments: the overall lesion segments, the GGO segments, and the consolidation segments. These image-level segments act as masks in the Python radiomic package PyRadiomics [41] for extracting image phenotypes, separately. Three runs of phenotype extraction are executed with the inputs of overall lesion segments plus the original images, the GGO segments plus the original images, and the consolidation segments plus the original images, respectively. We select first order measurements, such as Gray Level Co-occurrence Matrix (GLCM) measurement, Gray Level Dependence Matrix (GLDM) measurement, and Neighboring Gray Tone Difference Matrix (NGTDM) measurement, as our image phenotypes. The definition and formulas of these image phenotypes can be found in Additional file 1: Table S1. After the segments-based image-level phenotypes are generated, we take the average of them to make the image phenotypes at patient-level.

### Mediation analysis

Univariate mediation analyses are performed to determine the potential causal mechanism in which age, gender, or underlying diseases is associated with COVID-19 severity through an intermediate image phenotype. Let *y* be the dependent variable which is the binarized COVID-19 severity. In the original ICTCF dataset, severity is measured with 9 levels: Control (Healthy), Control, Control (Community-acquired pneumonia), Suspected, Suspected (COVID-19-confirmed later), Mild, Regular, Severe, and Critically ill. We code these 9 levels of the severity into 2 levels by grouping Control (Healthy), Control, Control (Community-acquired pneumonia), Suspected into one group (coded as 0) and Suspected (COVID-19-confirmed later), Mild, Regular, Severe, and Critically ill into another group (coded as 1). Let *m* be a mediator (patient-level image phenotype), *x* be an independent variable (age, gender, or underlying diseases). Hence, we can fit the below regression models [12]:

14 $$\begin{gathered} y = \beta_{10} + \beta_{11} x + \in_{1} \hfill \\ m = \beta_{20} + \beta_{21} x + \in_{2} \hfill \\ y = \beta_{30} + \beta_{31} x + \beta_{32} m + \in_{3} \hfill \\ \end{gathered}$$

Here, $\beta {\rm ~and~} \epsilon$ are the parameters of the models to be estimated and tested. $\beta s$ are the coefficients of variables, while $\epsilon$ are the residuals. If the abovementioned three regressions are significant (adjusted p-value < 0.05) and $\left|{\beta }_{11}\right|>|{\beta }_{31}|$, we say that *x* is associated with *y*, mediated through *m*, which provides a potential mechanism explanation of how *x* has influence on *y* through *m*. The indirect effect of *x* on *y* through m is defined as ${\beta }_{21}\times {\beta }_{32}$.

For multiple mediation analysis, we first perform a pair-wise correlation analysis of the significant mediators from the univariate mediation analysis using the R package, corrplot [42], to control the potential confounding influence on the multiple mediation analysis. The mediator pairs that have absolute correlation coefficient greater than 0.8 are first identified. Then, one phenotype within each of these correlated pairs is removed. The filtering criteria include both less indirect effect or less commonly used in medical research. The remaining mediators with the two independent variables (age and the underlying diseases) are input into a multiple mediation model for further identifying the indirect effect when controlling for each other using R package lavaan [43. Lavaan is a tool for structure equation modeling (SEM) which is a very general and powerful multivariate technique. SEM uses conceptual model, path diagram and linked regression-style equations to model complex relationships among a network of variables. Thus, it allows multiple independent variables and mediators, even multiple dependent variables in the model [43–45]. We build our equation system as below for our special case (two independent variables, one dependent variable, and several mediators linked to different independent variables.):

15 $$\begin{gathered} y = \theta_{00} + \theta_{01} x_{1} + \theta_{02} x_{2} + \varepsilon_{0} \hfill \\ M_{c} = \theta_{c0} + \theta_{c1} x_{1} + \theta_{c2} x_{2} + \varepsilon_{c} \hfill \\ M_{a} = \theta_{a0} + \theta_{a1} x_{1} + \varepsilon_{a} \hfill \\ M_{u} = \theta_{u0} + \theta_{u2} x_{2} + \varepsilon_{u} \hfill \\ y = \theta_{(n + 1)0} + \theta_{(n + 1)1} x_{1} + \theta_{(n + 1)2} x_{2} + \theta_{(n + 1)3} M_{1} + \ldots + \theta_{(n + 1)(n + 2)} M_{n} + \varepsilon_{n + 1} \hfill \\ \end{gathered}$$

where *x*_1_ is the age, *x*_2_ is the underlying disease. *M*_*c*_ is the significant mediators for both age and underlying disease. *M*_*a*_ represents the significant mediators for age, while *M*_*u*_ refers to the significant mediators for underlying diseases. The $\theta s$ are the coefficients which are estimated and tested when the model is fit. $\varepsilon$ are the residuals.

### Sensitivity analyses

A series of sensitivity analyses are performed to further support our conclusions. These analyses include: a three-fold cross validations performed using both single SSInfNet and multi SSInfNet to ensure that the performance is consistent, a comparison with transfer learning- based FCN8 segmentation network [46], further experiments on other independent datasets [47] to show the generalization ability of our models, ablation studies to explore which techniques (generative adversarial image inpainting, focal loss, and lookahead optimizer) we use in the multi SSInfNet model contribute to the improved performance, and a computation cost analysis to show the difference between the different models’ computation efficiency. The details of these analyses could be found in Additional file 1: Sensitivity Analysis.

## Results

### Single SSInfNet

**Fig. 3:**
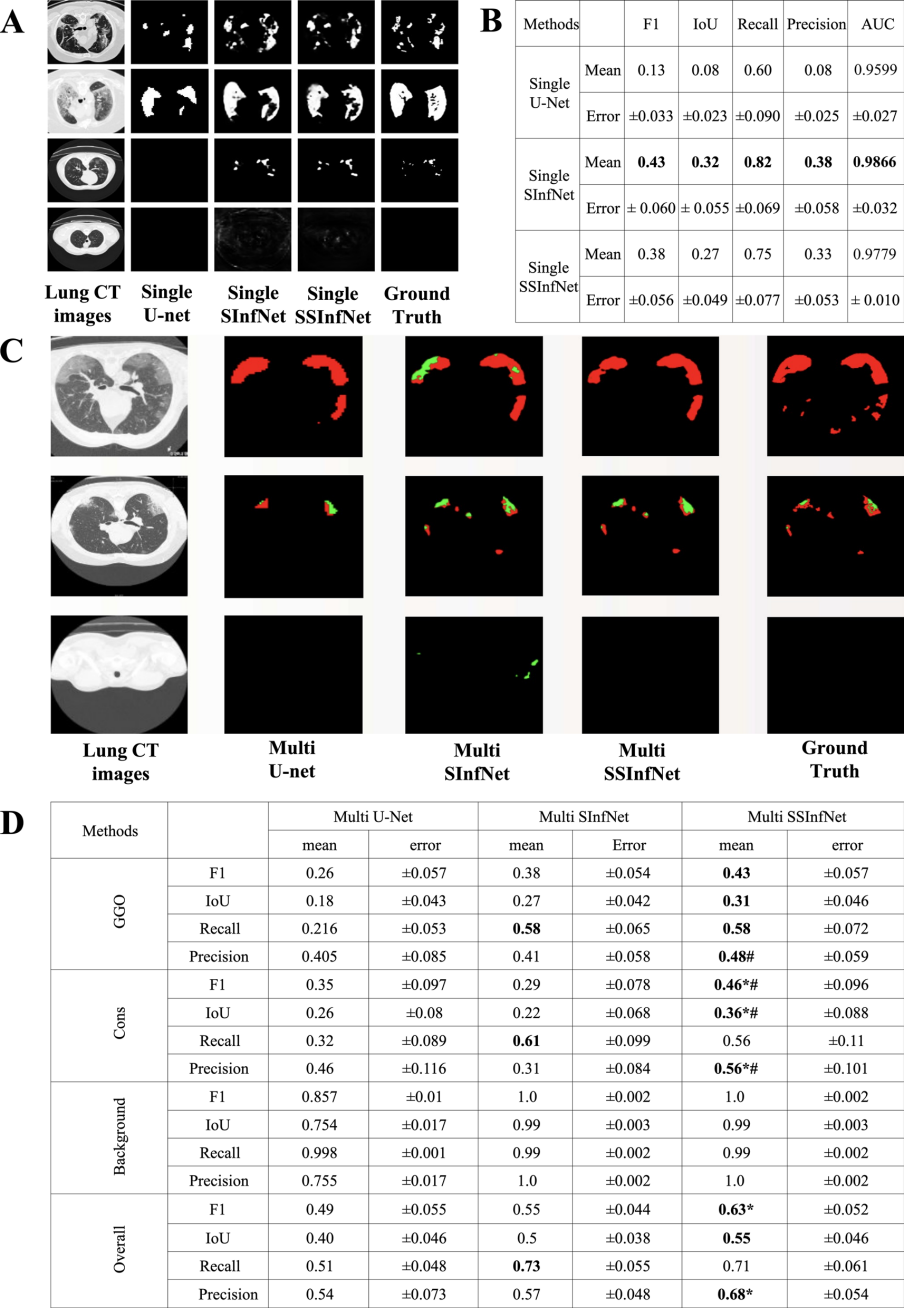
Visual comparison and quantitative comparison of segmentation results among different networks. **A** Four examples of the original lung CT images, their overall segments predicted by three different networks and the ground truth overall lesion annotation. The two baseline models are the single U-net and the single SInfNet (supervised COVID-19 lung infection segmentation) model. The proposed model is the single SSInfNet (self-supervised COVID-19 lung infection segmentation) model. **B** The mean and error of five quantitative model performance metrices calculated from the 35 test samples. **C** Three examples of the original lung CT images, their GGO and consolidation segments predicted by three different networks and the ground truth lesion annotations. The two baseline models are the multi U-net and the multi SInfNet models. The proposed model is the multi SSInfNet model. **D** The mean and error of five model performance metrics calculated from the 35 test samples. The Overall showed the averaged performance for GGO, consolidation, and background

The segmentation performance of the proposed single SSInfNet and the two baseline models (single U-net and single SInfNet) can be found in Fig. 3A and B. In this stage, the models do not segment either GGO or consolidation. They segment and represent the entire infected region as one overall lesion. U-Net [48] and supervised InfNet [22 (SInfNet) were selected as baseline models for comparing performance with our proposed SSInfNet. U-Net is a classical CNN and is often used as baseline or backbone of segmentation networks [49–53], while the SInfNet is our backbone model and was developed to solve the same COVID-19 segmentation problem. Five classical metrices (F1, IoU, Recall, Precision and AUC of the receiver operating characteristic) were used to quantitatively measure the networks’ performances. As the prediction is at the pixel level, we calculated the performance metrices at the sample level instead of the entire test set. Therefore, the mean and error for each of these performance metrices in the entire test set were shown in Fig. 3B. Observed from Fig. 3B**,** the proposed single SSInfNet and the baseline single SInfNet achieved comparable performances. The overall AUC and error based on the single SSInfNet is comparable to that of the single SInfNet (Additional file 1: Figures S3B, S4; Table S2), and both models outperform the baseline single U-net (Additional file 1: Figure S4) in terms of the overall AUC.

Even though the baseline single SInfNet has better mean values for F1, IoU, and Recall, the self-supervised approach helps create robustness and consistency in the model to better handle outliers. This can be observed by the fact that in Fig. 3A, the baseline single SInfNet overestimated the overall infected region of an outlier image (the last row) while the single SSInfNet did a better job at predicting outliers, where its prediction is more closely related to the ground truth than that of the baseline single SInfNet.

Even though the baseline single SInfNet has better mean values for F1, IoU, and Recall, the self-supervised InfNet approach helps handle some outliers in a better way. This can be observed by the fact that in Fig. 3A, the baseline single SInfNet overestimated the overall infected region of an outlier image (the last row) while the single SSInfNet did a better job at predicting outliers, where its prediction is more closely related to the ground truth than that of the baseline single SInfNet.

### Multi SSInfNet

Figure 3C and D show the multi SSInfNet performance. This is the second stage of solving the proposed multi segmentation problem. In this stage, the network breaks down the previous overall segments predicted by the single SSInfNet into two parts, the GGO and the consolidation. The overall segments from the single SSInfNet act as a prior and is fed, along with the CT lung images, into the multi SSInfNet. The output is a pixel level 3-channel matrix with each cell in one channel annotating the probability of being GGO, another channel annotating the probability of being consolidation, and the last channel annotating probability of being background. Again, the multi U-net and multi SInfNet were used as baseline models for comparison. The proposed multi SSInfNet was able to achieve better performance than the baseline multi U-net and multi SInfNet. As visualized in Fig. 3C, the multi SSInfNet achieved better performance in evaluating the GGO and the consolidation areas of the CT lung images than the multi SInfNet and the multi U-net, in terms of predicting the visible most similar segments to the ground truth. However, as Fig. 3D shows, the baseline multi SInfNet achieved a better recall than the rest of the networks. But, as we can see in Fig. 3C, multi SInfNet predicted more consolidation segments, even in areas that were not infected. On the third row of Fig. 3C, the multi SInfNet overestimated the consolidation region in a CT lung image from a healthy individual. Since recall is the true positives over the total actual consolidation area, the multi SInfNet seems to overestimate the consolidation area which results in a higher recall than the other networks. This explains well why the precision for the SInfNet is lower, as most of its prediction of the consolidation area is not accurate. Hence, this decreases the performance of the multi SInfNet while the proposed multi SSInfNet handled this problem very well.

### Mediation analysis

A total of 204 averaged image phenotypes for 1338 patients were created from the output of the proposed multi SSInfNet using Python’s radiomic package PyRadiomics [41]. The PyRadiomic algorithm needs a mask and the original image as input, and it outputs a list of continuous measures as image phenotypes. According to different measure approach, these image phenotypes can be categorized into first order features, Gray Level Co-occurrence Matrix (GLCM) features, Gray Level Dependence Matrix (GLDM) features, Neighboring Gray Tone Difference Matrix (NGTDM) features and so on. Three kinds of image segments (GGO, consolidation, and overall lesion) were separately input into the PyRadiomic algorithm as the mask of the original image. Each mask contributed 68 image phenotypes in four of the above defined phenotype categories, as listed in Additional file 1: Table S1.

**Fig. 4:**
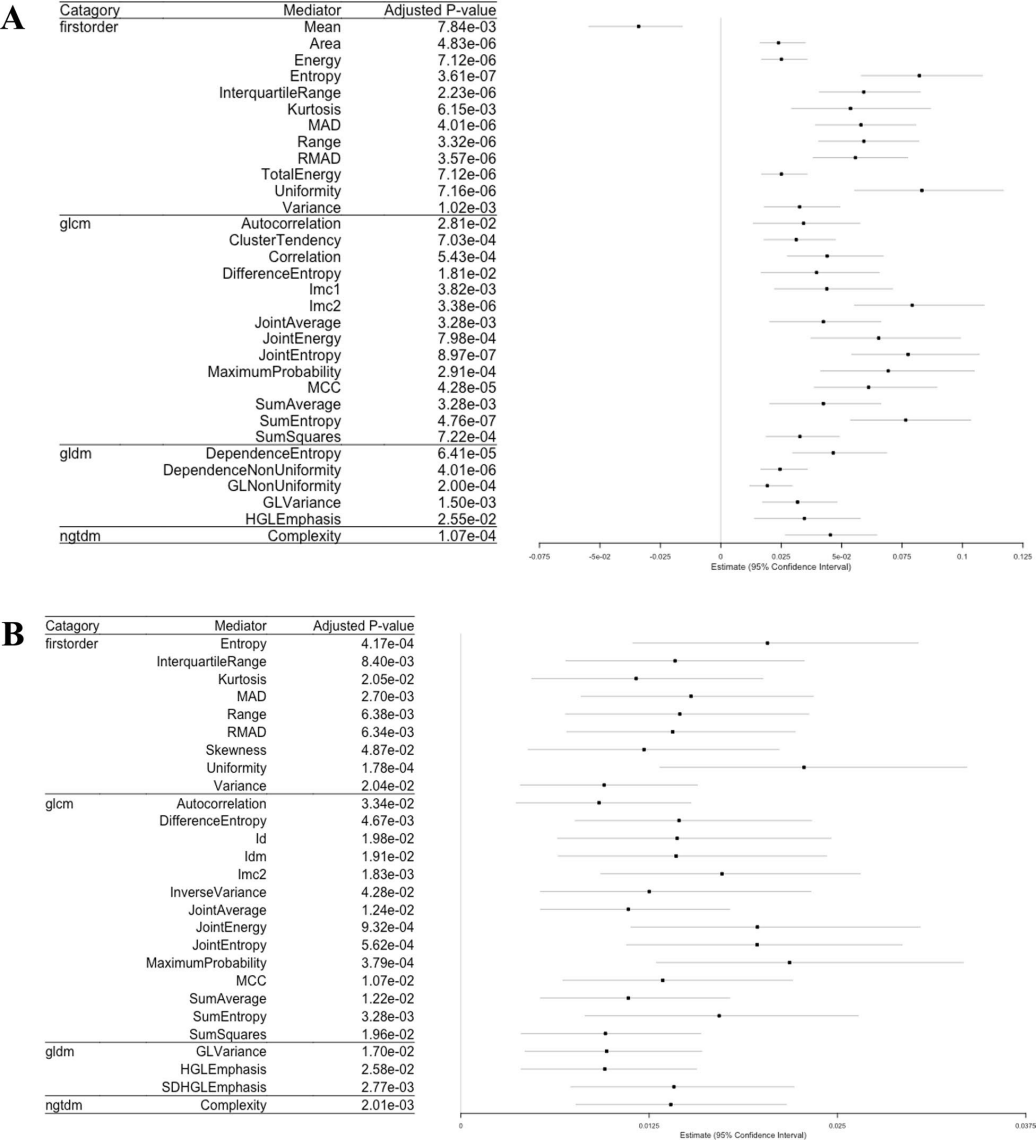
Significant image phenotypes in the univariate mediation analyses. **A** Forest plot showing the 32 mediators of age’s indirect effect on COVID-19 risk. **B** Forest plot showing the 27 mediators of underlying disease’s indirect effect on COVID-19 risk

R’s package, lavaan, was used to conduct the univariate mediation analysis for each combination of a given independent variable, dependent variable, and image phenotype. The independent variables include age (continuous), gender (binary), and the number of underlying diseases (continuous). The dependent variable is the binarized COVID-19 severity. All consolidation-based image phenotypes were not significant (adjusted p-value > 0.05) in the univariate mediation analysis. Therefore, the results of the consolidation- and overall lesion (consolidation + GGO)- based image phenotype analyses are not reported here. Among all 68 GGO- based image phenotypes, 37 of them have significant (adjusted p-value < 0.05) mediation effects on COVID-19 severity. Thirty-two of these 37 mediators were mediating the indirect effect of underlying disease on COVID-19 severity, while 27 of these 37 mediators were mediating the indirect effect of age on COVID-19 severity. The results, including the categories and names of the image phenotypes, the adjusted p-value of the indirect effects and the estimated coefficients for these image phenotypes, can be found in Fig. 4A (for age variable) and Fig. 4B (for underlying disease variable), respectively.

**Fig. 5:**
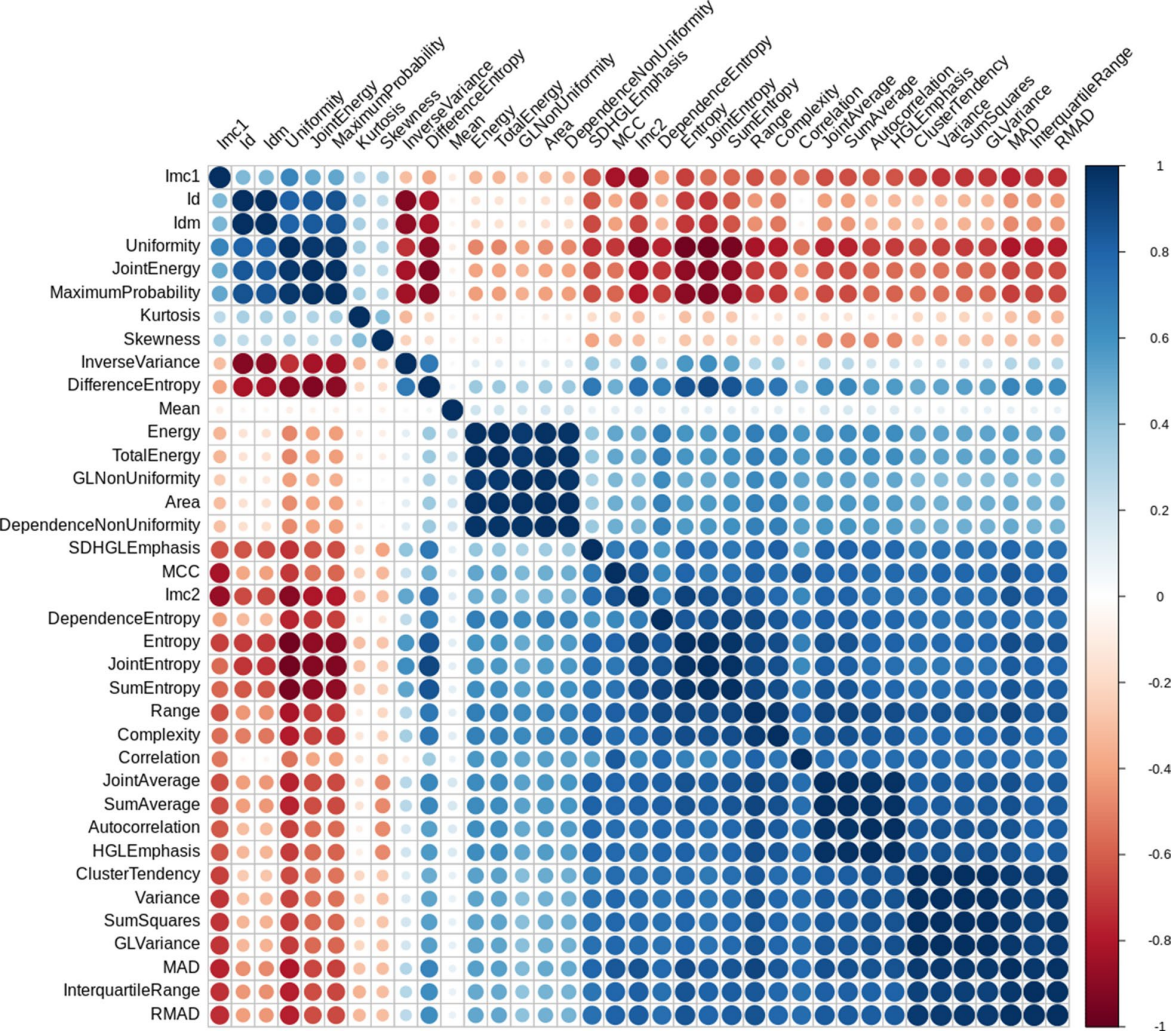
Hierarchical clustering of the ordered correlation matrix of the 37 image phenotypic mediators from the univariate mediation analysis. The color represents the correlation coefficient

**Fig. 6:**
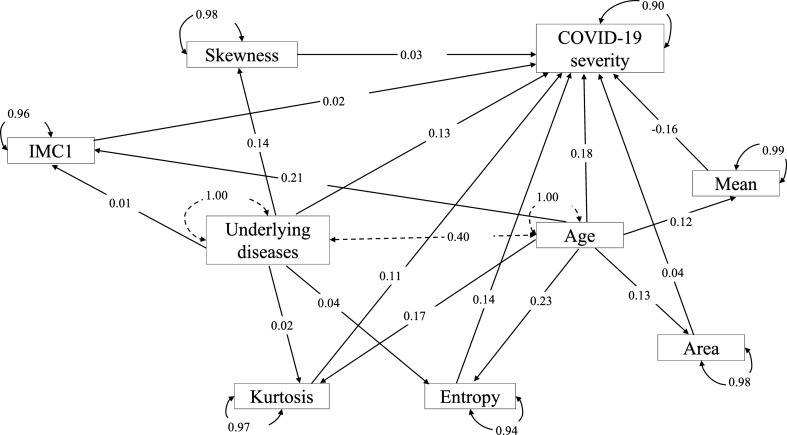
Path plot of the mediation analysis model with multiple mediators. The standardized effect estimates of each variable are shown on the edges of the paths. The mediators are Entropy, Kurtosis, Skewness, Mean, Area, and IMC1. Dependent variable is the COVID-19 severity, and independent variables are the Underlying disease and Age. Curves with arrowheads on both sides is the standardized residual variance. Solid curve is for dependent variable and mediators. Dash line curve is for independent variables. Straight dash line represents the standardized covariance of two independent variables. Straight solid line with arrowhead on one side is the standardized effect estimate

The correlations among these mediators were calculated based on their patient-level averaged feature values, and the results are demonstrated in Fig. 5. Mediators with high absolute correlation coefficients are more linearly dependent and hence have similar effect on COVID-19 severity. So, the correlation between each pair of the image phenotypes was compared and one of the two phenotypes was removed if their absolute correlation coefficient was greater than 0.8. It was suggested by the PyRadiomics that some features are the confounding of the segment area [41. Thus, only the area variable was kept among those phenotypes. Since the entropy measures the uniformity and is more widely incorporated in medical image phenotype related researches [54], the entropy phenotype was kept while the uniformity phenotype was removed. The MCC (Maximal Correlation Coefficient) and IMC1 (Informational Measure of Correlation) measures the complexity of the texture. We decided to keep IMC1 because it has been widely used and reported in lung cancer studies [55, 56]. After the correlation filtering, 3 mediators (Entropy, Kurtosis, and Skewness) were left for the underlying disease variable and 5 mediators (Mean, Area, Entropy, Kurtosis, and IMC1) were left for the age variable. The remaining 3 and 5 mediators were input into the mediation analysis models with multiple mediators. The results can be found in the path plot (Fig. 6).

### Sensitivity analyses

From the results of the three-fold cross-validation (Additional file 1: Table S2), we can see that the baseline single SInfNet performs the best for most of the performance metrics. Self-supervised SSInfNet does not show an improvement in the single segmentation for the CT lung images. However, as shown in Additional file 1: Table S3, the multi SSInfNet shows a better performance than both the multi U-Net and the multi SInfNet in the three-fold cross-validation, suggesting that the multi SSInfNet can generalise well in segmenting GGO and consolidation regions of the CT lung images. These three-fold cross-validation based results are consistent with those shown in Fig. 3B (single InfNet) and 3D (multi InfNet) based on the training, test, and validation strategy used to develop the models.

As shown in the Additional file 1: Table S4, the proposed new method, multi SSInfNet, achieves the best performance among the multi FCN8 (with pre-trained weights), mullti U-Net, and the baseline multi SInfNet models. Further experiments on other independent datasets were applied to evaluate the generalization ability of our models (Additional file 1: Tables S5, S6). For the independent data set 2 (see Additional file 1: Additional data sets), due to the nature of the very small dataset on which we tested the methods, the dataset did not replicate a good generalisation behaviour from the methods that were trained on (Additional file 1: Table S5A (Single InfNet) and S5B (Multi InfNet)). However, for a relatively larger independent data set 3 (see Additional file 1: Additional data sets), we can see that its results (Additional file 1: Table S6B) are consistent with our current dataset where our multi SSInfNet shows a better performance in segmenting the CT lung images (Fig. 3D) than the baseline multi SInfNet (Fig. 3B). The results that we obtained from the baseline SInfNet and the SSInfNet can be found in Additional file 1: Table S6A.

The results of the ablation studies can be found in Additional file 1: Table S7. We can see that all the additional techniques added on the baseline SInfNet have improved performance on the segmentation of the CT lung images. They compensate with each other and then achieve a higher performance.

The computational cost analysis of different models is shown in the Additional file 1: Table S8. Overall, the time taken to process 1 image for baseline multi SInfNet is 1.06 times longer than the time taken to process 1 image for multi SSInfNet. The multi FCN8 has the best computation efficiency with 0.74 times shorter time than the time taken by the baseline multi SInfNet.

## Discussion

Lung CT imaging was proposed as a backup diagnosis and monitoring tool for COVID-19 in emergency breakouts when PCR kits are not available [57, 58]. Others also suggested that PCR testing and lung CT imaging should be used together for routine COVID-19 diagnosis and prognosis to enhance the clinical protocol of COVID-19 [58, 59]. Some radiologists suggested to avoid using confirmative statement about COVID-19 identification from lung CT imaging because CT may not be able to distinguish among different viruses [60], but a recent study showed that 7 radiologists successfully identified COVID-19 distinguished from other viral infections with 93–100% specificity based on lung CT imaging features [61]. In many studies, lung CT imaging showed comparable sensitivity with PCR in diagnosis of COVID-19. The sensitivity of PCR ranges from 42 to 71% [57, 60, 62], while the reported sensitivity of lung CT imaging-based diagnosis for COVID-19 varies from 60 to 90% [57, 60, 62–64]. There are also some concerns about the cost of lung CT imaging as the PCR test is much cheaper than lung CT scan [65]. Although the cost of healthcare services is complex, the major cost of a medical imaging-based test is spent on using radiologists for image reading [66]. Hence, it is a promising area to introduce AI into lung CT imaging-based diagnosis to assist radiologists. Furthermore, it is possible to increase the sensitivity and specificity of PCR alone or human involved lung CT imaging COVID-19 diagnosis.

When introducing AI into healthcare, the accuracy of the model is not the whole picture. The interpretability and transparency of the model should always be kept in mind. Lung CT image segmentation, in its very core spirit, is to split human understandable regions from the less informative background regions to assist people’s decision-making. Therefore, lung CT image segmentation model makes more clinical sense than a binary classifier which takes the raw lung CT image as input and gives a simple yes or no answer especially. The computationally segmented lung regions could be further analyzed for biological explanations and diagnostic values.

InfNet is able to achieve a competent performance on segmentation of infected region for CT lung images. The authors of the InfNet further extended the network by introducing semi-supervised learning to InfNet. They generated pseudo labels and utilized the pseudo labels to train their model using a two-step strategy. However, their method of semi-supervised by generating pseudo-labels when used in the dataset that we used takes a few months to finish generating the pseudo-labels. This is not feasible in real world applications. Hence, here we propose to use a self-supervised learning strategy. The self-supervised method creates a huge speed up improvement when compared to their method of semi-supervised learning.

To increase the model performance, self-supervised image inpainting was used in this study for model pre-training, focal loss was used to replace the traditional cross entropy loss, Lookahead optimizer was used along with SGD to manage the training iteration. The integration of these advanced techniques achieved the best model performance, as compared to other baseline models. The proposed Multi SSInfNet model is better at dealing with outliers and makes fewer false positive in predicting the minority class, which, for COVID-19, is the consolidation lesion.

To enhance the interpretation of the proposed Multi SSInfNet model, we further extracted the lung imaging phenotypes from the output of the model and applied statistical mediation analysis to explore the potential causal association of the patients’ age, gender, and underlying diseases with COVID-19 severity through the identified lung CT imaging mediators. We showed that 8 image phenotypes from the predicted GGO segments were significantly correlated with COVID-19 severity and the age or underlying disease(s) of a patient with COVID-19. The entropy and kurtosis of the computational GGO segments have a positive mediation effect on both underlying diseases caused COVID-19 severity and age caused COVID-19 severity. Entropy represents the uncertainty of the pixel values within the GGO segment. A higher entropy indicates a more chaotic GGO segment [41]. Kurtosis measures the peakedness of the GGO pixel value distribution. A higher kurtosis implies that the GGO pixel values are concentrated towards the tails rather than towards the mean [41]. This means that elders or people with sever underlying diseases will probably have more chaotic and peakier distributed GGO segments, and thus will suffer from severer COVID-19. In addition to these two lung image phenotypes, the area and IMC1 of the computational GGO segments have a positive mediation effect on age caused COVID-19 severity, which suggests that elders often have bigger GGO lesions and more correlate distributed probability of the pixel values [41] within the GGO regions. Skewness of computational GGO segments also has a positive mediation effect on underlying diseases caused COVID-19 severity, which suggests that underlying diseases could cause asymmetrically distributed pixel values within GGO regions, thus leading to severer COVID-19. Interestingly, these lung image phenotypes have also been reported as potential image biomarkers for lung adenocarcinoma [67, non-small cell lung cancer [68], pulmonary interstitial pneumonia [69], and so on [70, 71].

One limitation of this study is that we do not have the resource to recruit radiologists into our experiment. We also do not have failure and success information of the PCR test and lung CT image test for the same patients. These could be future directions for institutes that are able to get these resources.

## Conclusion

In conclusion, our work carefully considers several aspects of AI-based COVID-19 imaging diagnosis and prognosis, in terms of the model performance, model interpretability, and biological mechanism of the computational segmental image phenotypes associated with COVID-19. A series of sensitivity analyses have shown the robustness and generalizability of our proposed method. The clinical explanation of the computational GGO segment is also well addressed. Eight GGO segment-based image features have been identified as potential image biomarkers for COVID-19 severity. Comparing with previous works, our model shows better performance and is well interpreted both clinically and statistically.

## Supplementary Information

**Additional file 1**: **Figure S1**. Architecture of the supervised InfNet. **Figure S2**. A is the original architecture of the SInfNet. B is the architecture of our self-supervised InfNet model. Highlighted purple block is the difference between the original single SInfNet and the single SSInfNet. **Figure S3**. A is the architecture of the original multi SInfNet model. B is the architecture of our self-supervised multi InfNet model. Highlighted green block is the difference between the original multi SInfNet and our self-supervised multi SSInfNet. **Figure S4**. ROC for single InfNet. **Algorithm S1**. SSInfNet. **Table S1**. Image phenotypes. **Table S2**. The three-fold cross-validation performance of single networks. It should be noted that the data were obtained by combining the training, testing, and validation set from the Med-Seg (medical segmentation) COVID-19 dataset, and then splitting the combined data into 3 folds. **Table S3**. The three-fold cross validation performance of multi networks. **Table S4**. Comparison with transfer learning based FCN8 network. Quantitative result of Ground-glass Opacities & Consolidation on the test data set of the Med-Seg (medical segmentation) COVID-19 dataset. Prior was obtained from the single segmentation InfNet. **Table S5**. Model performance on independent COVID-19 CT Dataset set 2. **Table S6**. Model performance on the independent COVID-19 CT Data set 3. **Table S7**. Results of ablation studies. The performance of the ablation of our proposed multi-SSInfNet. Multi-SSInfNet refers to the self-supervised SInfNet with Focal Loss and Lookahead optimizer. We tried a variety of the model with a subtraction of the different technologies to carry out the ablation. **Table S8**. Computational costs of processing one image

## Data Availability

Code for SSInfNet is available at https://github.com/darylfung96/self-supervised-CT-segmentation.

## Abbreviations

AI: Artificial intelligence
AUC: Area under the curve
CNN: Convolutional neural network
CT: Computerized tomography
GAN: Generative adversarial network
GGO: Ground-glass opacity
GLCM: Gray level co-occurrence matrix
GLDM: Gray level dependence matrix
ICTCF: Integrative resource of lung CT images and clinical features
InfNet: COVID-19 lung infection segmentation network
IoU: Intersection over union
Med-Seg: Medical segmentation
NGTDM: Neighboring gray tone difference matrix
PCR: Polymerase chain reaction
ROC: Receiver operating characteristic
SE: Standard error
SEM: Structure equation modeling
SGD: Stochastic gradient descent
SInfNet: Supervised COVID-19 lung infection segmentation network
SSInfNet: Self-supervised COVID-19 lung infection segmentation network
