# Supplementary material for: Self-supervised deep learning model for COVID-19 lung CT image segmentation highlighting putative causal relationship among age, underlying disease and COVID-19
Source: J Transl Med. 2021 Jul 26;19:318. doi: 10.1186/s12967-021-02992-2 (PMC8312213; doi:10.1186/s12967-021-02992-2)
Supplement: Supplementary file 1 — Additional file 1: Figure S1. Architecture of the supervised InfNet. Figure S2. A is the original architecture of the SInfNet. B is the architecture of our self-supervised InfNet model. Highlighted purple block is the difference between the original single SInfNet and the single SSInfNet. Figure S3. A is the architecture of the original multi SInfNet model. B is the architecture of our self-supervised multi InfNet model. Highlighted green block is the difference between the original multi SInfNet and our self-supervised multi SSInfNet. Figure S4. ROC for single InfNet. Algorithm S1. SSInfNet. Table S1. Image phenotypes. Table S2. The three-fold cross-validation performance of single networks. It should be noted that the data were obtained by combining the training, testing, and validation set from the Med-Seg (medical segmentation) COVID-19 dataset, and then splitting the combined data into 3 folds. Table S3. The three-fold cross validation performance of multi networks. Table S4. Comparison with transfer learning based FCN8 network. Quantitative result of Ground-glass Opacities & Consolidation on the test data set of the Med-Seg (medical segmentation) COVID-19 dataset. Prior was obtained from the single segmentation InfNet. Table S5. Model performance on independent COVID-19 CT Dataset set 2. Table S6. Model performance on the independent COVID-19 CT Data set 3. Table S7. Results of ablation studies. The performance of the ablation of our proposed multi-SSInfNet. Multi-SSInfNet refers to the self-supervised SInfNet with Focal Loss and Lookahead optimizer. We tried a variety of the model with a subtraction of the different technologies to carry out the ablation. Table S8. Computational costs of processing one image [file 12967_2021_2992_MOESM1_ESM.pdf]

Self-supervised Deep Learning Model for COVID-19 Lung CT Image Segmentation Highlighting Putative Causal Relationship among Age, Underlying Disease and COVID-19

Daryl L.X. Fung, Qian Liu, Judah Zammit, Carson Kai-Sang Leung, Pingzhao Hu

Sensitivity Analyses

A series of sensitivity analyses are performed to further support our conclusions. These analyses include: three-fold cross validations using both single SSInfNet and multi SSInfNet to ensure that the performance is consistent, a comparison with transfer learning- based FCN8 (fully convolutional neural network architecture) segmentation network[1], further experiments on other independent datasets[2] to show the generalization ability of our models, ablation studies to explore which techniques (generative adversarial image inpainting, focal loss, and lookahead optimizer) we used in the multi SSInfNet contribute to the improved performance, and a computation cost analysis to show the difference between the different models' computation efficiency. The details of these analyses could be found below.

1. Three-fold cross-validation

We carried out a three-fold cross-validation on the Med-Seg (medical segmentation) COVID-19 Dataset as shown in **Figure 2D** to test the robustness of the proposed SSInfNet. We did this for both single SSInfNet and multi SSInfNet. Since the analysis is time consuming, we did not perform five-fold or 10-fold cross-validation analysis. During self-supervision, we trained the multi SSInfNet to reconstruct the CT lung images and the prior by replacing the last layer to output the reconstruction of the CT lung images. As for the self-supervision of single SSInfNet, we trained

the single SSInfNet to reconstruct the edge and the CT lung images. We undergo self-supervision to help the single SSInfNet and multi SSInfNet learn a good representation of the CT lung images before transferring the learned weights to train on segmenting the infected region of the CT lung images to determine if there is an improvement in performance.

2. Comparison with transfer learning

To address the data set with small labeled samples, we also carried out a comparison of our method and the baseline method with a transfer learning technique, which is also frequently used to overcome small sample size issue [1]. We compared against FCN8 network for segmenting the CT lung images in the Med-Seg (medical segmentation) COVID-19 Dataset as shown in **Figure 2D**. We transferred the learned weights from VGG16 network to the multi FCN8 network and started the training from the pre-trained weights. We then compared the performance of multi FCN8 network with the baseline multi SInfNet and the multi SSInfNet. Originally, the multi FCN8 network receive 3 input channels, we changed the input channels to be 6 to make the model consistent with the other model where the model receives the prior and the CT lung images of which both are concatenated together to form 6 input channels. For the multi SSInfNet, the focal loss alpha is set as 1 and the gamma is set as 2, the lookahead optimizer k is set as 5 and the alpha is set as 0.5. All other parameters are kept the same.

3. Additional independent data sets

To further compare the performance of our proposed method with other baseline methods, we tested them on two additional data sets, which are called as Data set 2 and Data set 3, respectively.

The Med-Seg (medical segmentation) COVID-19 Data set as shown in **Figure 2D** is called as Data set 1.

The Data sets 2 and 3 are detailed as follows: **Data set 2**: This is the original dataset that was used to evaluate the SInfNet [2]. It contains 50 single labeled CT lung images and 48 multi labeled CT lung images for the training set; 48 single & multi labeled images for testing set. There is no validation set. **Data set 3**: The dataset contains 750 CT images for which the segmentation mask is available[3]. These come from 150 patients with novel-coronavirus pneumonia. The images were labelled by a panel of five senior radiologist with over 25 years of experience. The labels used were healthy lung-field, GGO and consolidation. We used the labelled CT images to train a U-Net semantic segmentation model that effectively segments the lung field present in the CT image. Using this model, as well as the opening and closing morphological transformations for noise reduction, we cropped the CT images so that they would only include lung field. Then, for efficiency reasons, we took the middle most slice of each CT scan and removed all others. This ensures that we have a data set with a similar amount of diversity to the original data set, while being significantly smaller. After this, we manually removed any CT images that did not have the lungs in full view or had a significant amount of non-lung field present in the CT image.

4. Ablation studies

We carried out ablation studies to compare the performance differences between the combination of the different techniques that we incorporated into the multi SSInfNet. This analysis helped us determine which one contributes to the improved performance. We carried out 4 different ablations

of our proposed Multi SSInfNet: Multi SSInfNet, Multi SSInfNet – focal loss (without focal loss), Multi SSInfNet – lookahead optimizer (without lookahead optimizer), Multi SSInfNet – focal loss – lookahead optimizer (without focal loss and lookahead optimizer). All other parameters are maintained the same with focal loss alpha as 1 and gamma as 2, the lookahead optimizer k value as 5 and alpha as 0.5.

5. Computation cost analysis

We performed a computation cost analysis to show the difference between the different models' computation efficiency.

Reference

1. Hooda R, Mittal A, Sofat S. Lung segmentation in chest radiographs using fully convolutional networks. Turkish J Electr Eng Comput Sci [Internet]. 2019 [cited 2021 Jun 29];27:710–22. Available from: https://journals.tubitak.gov.tr/elektrik/abstract.htm?id=24422

2. Fan D-P, Zhou T, Ji G-P, Zhou Y, Chen G, Fu H, et al. Inf-Net: Automatic COVID-19 Lung Infection Segmentation from CT Images. IEEE Trans Med Imaging [Internet]. Institute of Electrical and Electronics Engineers Inc.; 2020 [cited 2020 Sep 22];39:2626–37. Available from: http://arxiv.org/abs/2004.14133

3. Zhang K, Liu X, Shen J, Li Z, Sang Y, Wu X, et al. Clinically Applicable AI System for Accurate Diagnosis, Quantitative Measurements, and Prognosis of COVID-19 Pneumonia Using Computed Tomography. Cell. Cell Press; 2020;181:1423-1433.e11.

Supplementary Figures

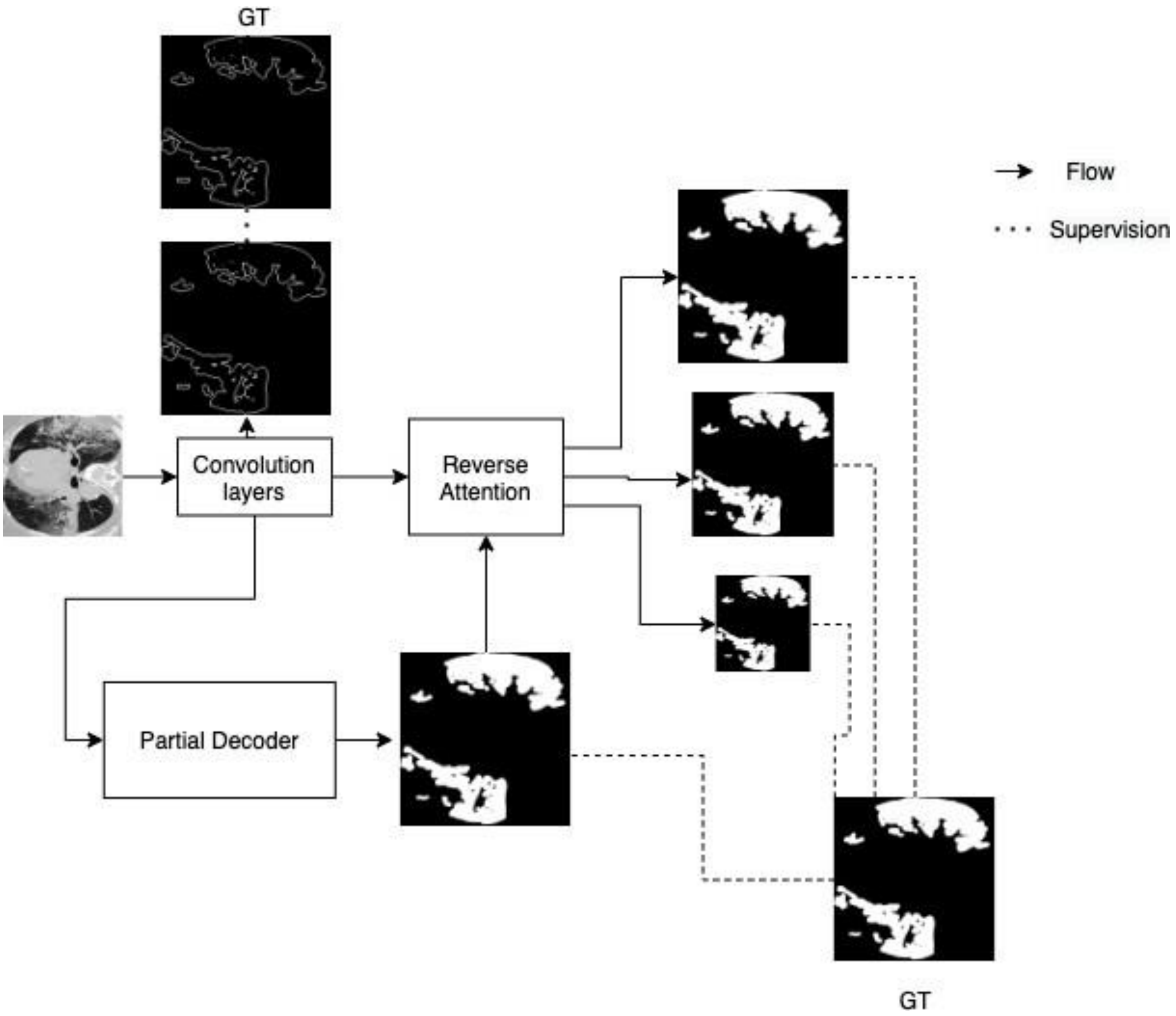

Supplementary Figure 1. Architecture of the supervised InfNet.

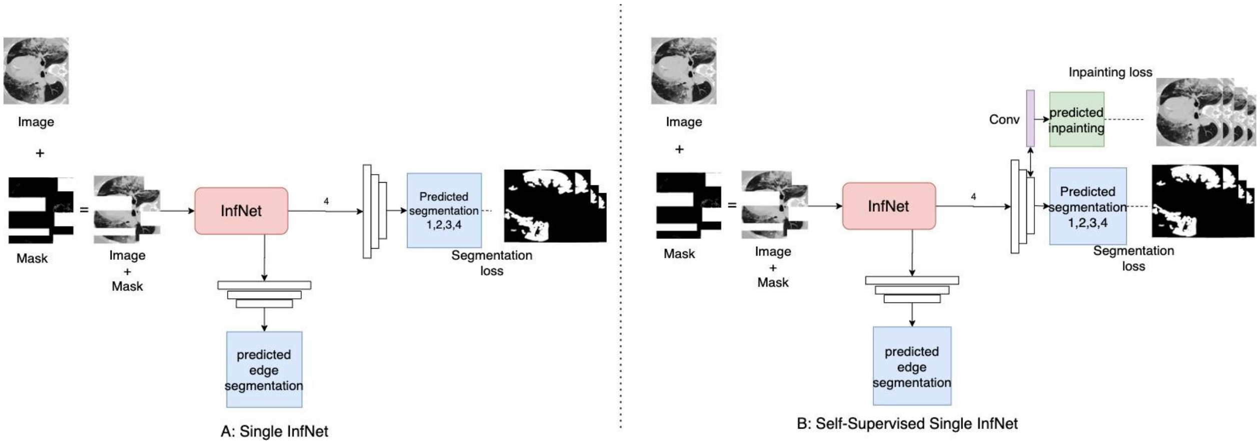

Supplementary Figure 2. A is the original architecture of the SInfNet. B is the architecture of our self-supervised InfNet model. Highlighted purple block is the difference between the original single SInfNet and the single SSInfNet.

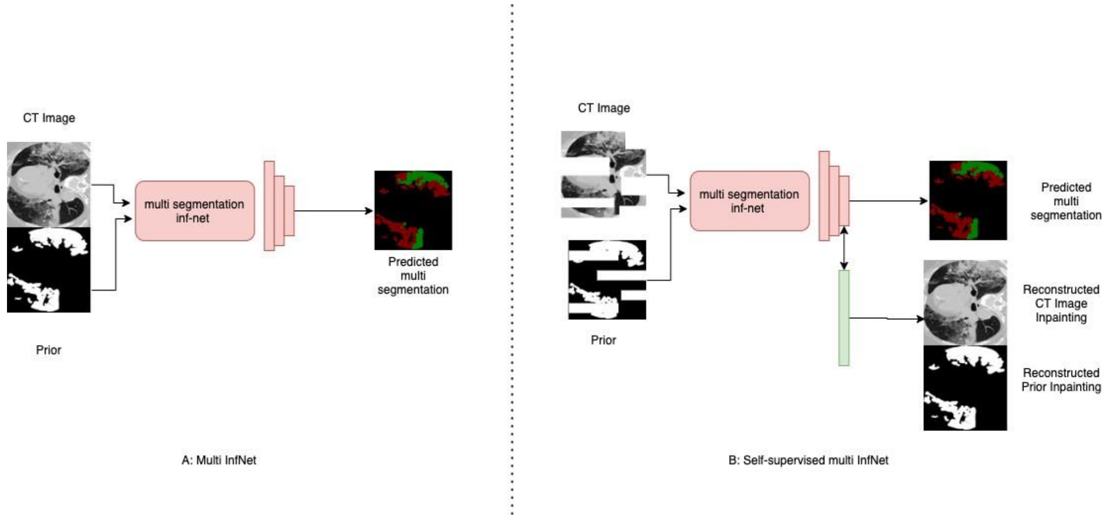

Supplementary Figure 3. A is the architecture of the original multi SInfNet model. B is the architecture of our self-supervised multi InfNet model. Highlighted green block is the difference between the original multi SInfNet and our self-supervised multi SSInfNet.

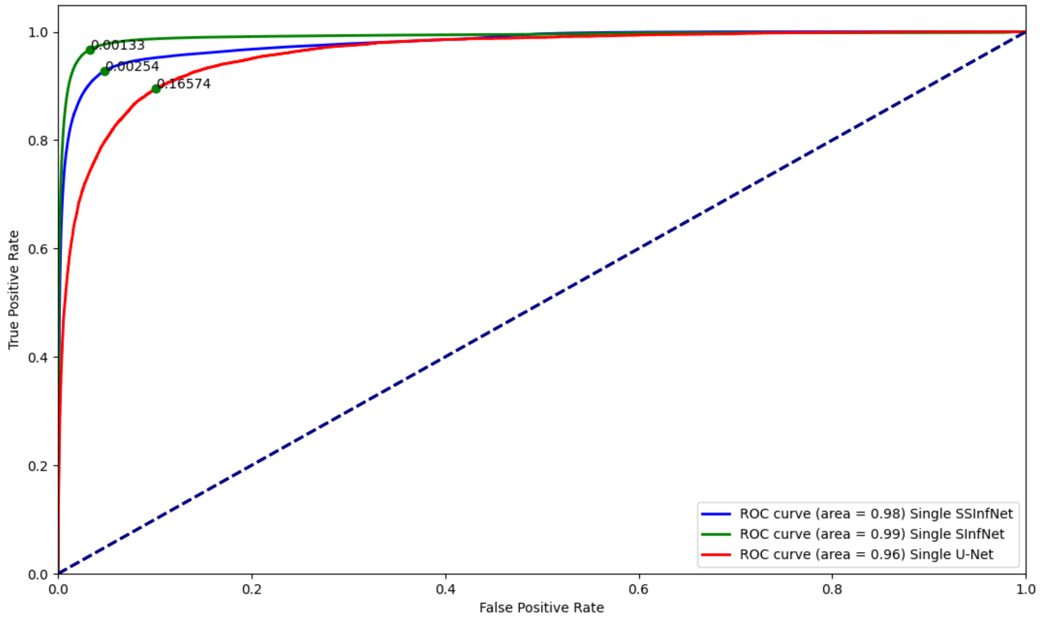

Supplementary Figure 4. ROC for single InfNet

Supplementary Algorithm 1. SSInfNet

Algorithm 1 Pseudo code for self-supervised with InfNet

Input: $D_{labeled}$ = [($inputImage_1$, G_{t1}), ...]
for each epoch **do**
  for each coach step **do**
    mask = M(x)
    maskedInput = $mask \odot inputImage$
    $predictedImage = network(maskedInput), inputImage$
    $L_{rec} = CrossEntropy(predictedImage, inputImage)$
    $L_{coach}(x) = 1 - L_{rec}$
    update coach weights
  end for
  for each network step **do**
    $P_{labeled} = Preprocess(D_{labeled})$
    $inpaintingOutput = network(P_{labeled})$
    $L_{rec} = CrossEntropy(InpaintingOutput, inputImage)$
    backpropogate and save network weights
  end for
end for
for each batch of $D_{labeled}$: **do**
  $P_{labeled}$ = Preprocess ($D_{labeled}$)
  $trainLoss = train(P_{labeled})$
  Backpropagate train loss
  $testLoss = test(P_{labeled})$
  save model weights, w.
end for

Supplementary Tables

Supplementary Table 1. Image phenotypes

Image Phenotype		Description	Formula
First Order Features (20)	Area	The number of pixels in the mask.	
	Energy	The magnitude of voxel values in an image.	$\sum_{i=1}^{N_p} (X(i) + c)^2$ Here, c is optional value shifting the intensities to prevent negative values in **X**
	Total Energy	Energy scaled by the volume of the voxel.	$V_{voxel} \sum_{i=1}^{N_p} (X(i) + c)^2$
	Entropy	The uncertainty/randomness in the image values.	$-\sum_{i=1}^{N_g} p(i) \log_2(p(i) + \epsilon)$ Here, ϵ is an arbitrarily small positive number ($\approx 2.2\times10^{-16}$).
	Minimum	The Minimum of **X**	min(**X**)
	10th percentile	The 10th percentile of **X**	
	90th percentile	The 90th percentile of **X**	
	Maximum	The maximum of **X**	max(**X**)
	Mean	The average gray level intensity.	$\frac{1}{N_p} \sum_{i=1}^{N_p} X(i)$
	Median	The median gray level intensity.	
	Interquartile Range	The subtract of 25th and 75th percentile of the image array.	**P**75−**P**25
	Range	The range of gray values.	max(**X**)−min(**X**)
	Mean Absolute Deviation (MAD)	The mean distance of all intensity values from the Mean Value of the image array.	$\frac{1}{N_p} \sum_{i=1}^{N_p} \lvert X(i) - \bar{X} \rvert$
	Robust Mean Absolute Deviation (RMAD)	The mean distance of all intensity values from the mean value.	$\frac{1}{N_{10-90}} \sum_{i=1}^{N_{10-90}} \lvert X_{10-90}(i) - \overline{X_{10-90}} \rvert$
	Root Mean Squared (RMS)	The square-root of the mean of all the squared intensity values.	$\sqrt{\frac{1}{N_p} \sum_{i=1}^{N_p} (X(i) + c)^2}$
	Skewness	The asymmetry of the distribution of values about the mean value.	$\frac{\frac{1}{N_p}\sum_{i=1}^{N_p}(X(i) - \bar{X})^3}{\sqrt{\frac{1}{N_p}\sum_{i=1}^{N_p}(X(i) - \bar{X})^2}^{\,3}}$

	Kurtosis	A higher value means that the mass of the distribution is concentrated towards the tail(s) rather than towards the mean. A lower value means that the mass of the distribution is concentrated near the mean value.	$\frac{\frac{1}{N_p}\sum_{i=1}^{N_p}(X(i)-\bar{X})^4}{\left(\sqrt{\frac{1}{N_p}\sum_{i=1}^{N_p}(X(i)-\bar{X})^2}\right)^4}$
	Variance	The mean of the squared distances of each intensity value from the Mean value.	$\frac{1}{N_p}\sum_{i=1}^{N_p}(X(i)-\bar{X})^2$
	Uniformity	A higher value means a smaller range of discrete intensity.	$\sum_{i=1}^{N_g} p(i)^2$
Gray Level Co-occurrence Matrix (GLCM) Features (28)	Autocorrelation	the magnitude of the fineness and coarseness of texture.	$\sum_{i=1}^{N_g}\sum_{j=1}^{N_g} p(i,j)i\,j$
	Joint Average	Returns the mean gray level intensity of the *i* distribution.	$\sum_{i=1}^{N_g}\sum_{j=1}^{N_g} p(i,j)i$
	Cluster Prominence	The skewness and asymmetry of the GLCM.	$\sum_{i=1}^{N_g}\sum_{j=1}^{N_g}(i+j-\mu_x-\mu_y)^4 p(i,j)$
	Cluster Shade	The skewness and uniformity of the GLCM.	$\sum_{i=1}^{N_g}\sum_{j=1}^{N_g}(i+j-\mu_x-\mu_y)^3 p(i,j)$
	Cluster Tendency	The grouping of voxels with similar gray-level values.	$\sum_{i=1}^{N_g}\sum_{j=1}^{N_g}(i+j-\mu_x-\mu_y)^2 p(i,j)$
	Contrast	The local intensity variation. A larger value is associated with a greater disparity among neighboring voxels.	$\sum_{i=1}^{N_g}\sum_{j=1}^{N_g}(i-j)^2 p(i,j)$
	Correlation	The linear dependency of gray level values to their respective voxels in the GLCM.	$\frac{\sum_{i=1}^{N_g}\sum_{j=1}^{N_g} p(i,j)ij-\mu_x\mu_y}{\sigma_x(i)\sigma_y(j)}$
	Difference Average	The difference between occurrences of pairs with similar intensity values and occurrences of pairs with differing intensity values.	$\sum_{k=0}^{N_g-1} kp_{x-y}(k)$
	Difference Entropy	The randomness/variability in neighborhood intensity value differences.	$\sum_{k=0}^{N_g-1} p_{x-y}(k)\log_2(p_{x-y}(k)+\epsilon)$
	Difference Variance	The heterogeneity that places higher weights on differing intensity level pairs that deviate more from the mean.	$\sum_{k=0}^{N_g-1}(k-DA)^2 p_{x-y}(k)$
	Dissimilarity		$\sum_{i=1}^{N_g}\sum_{j=1}^{N_g}\lvert i-j\rvert p(i,j)$
	Joint Energy	The homogeneous patterns in the image. A greater Energy implies	$\sum_{i=1}^{N_g}\sum_{j=1}^{N_g} p(i,j)^2$

		that there are more instances of intensity value pairs in the image.	
	Joint Entropy	The randomness/variability in neighborhood intensity values.	$-\sum_{i=1}^{N_g}\sum_{j=1}^{N_g} p(i,j)^2 \log_2(p(i,j)+\epsilon)$
	Homogeneity 1		$\sum_{i=1}^{N_g}\sum_{j=1}^{N_g} \frac{p(i,j)}{1+\lvert i-j\rvert}$
	Homogeneity 2		$\sum_{i=1}^{N_g}\sum_{j=1}^{N_g} \frac{p(i,j)}{1+\lvert i-j\rvert^2}$
	Informational Measure of Correlation 1 (IMC1)	The correlation between the probability distributions of *i* and *j* (quantifying the complexity of the texture), using mutual information I (x, y)	$\frac{HXY - HXY1}{\max\{HX, HY\}}$ $-I(i,j) = \sum_{i=1}^{N_g}\sum_{j=1}^{N_g} p(i,j)\log_2\left(p_x(i)p_y(j)\right) - \sum_{i=1}^{N_g}\sum_{j=1}^{N_g} p(i,j)\log_2(p(i,j)) = HXY - HXY1$
	Informational Measure of Correlation 2 (IMC2)	The correlation between the probability distributions of *i* and *j* (quantifying the complexity of the texture).	$\sqrt{1-e^{-2(HXY2-HXY)}}$
	Inverse Difference Moment (IDM)	The local homogeneity of an image.	$\sum_{k=0}^{N_g-1} \frac{p_{x-y}(k)}{1+k^2}$
	Maximal Correlation Coefficient (MCC)	The complexity of the texture	MCC = $\sqrt{\text{second largest eignvalue of Q}}$ $Q(i,j) = \sum_{k=0}^{N_g} \frac{p(i,k)p(j,k)}{p_x(i)p_y(k)}$
	Inverse Difference Moment Normalized (IDMN)	The local homogeneity of an image.	$\sum_{k=0}^{N_g-1} \frac{p_{x-y}(k)}{1+(\frac{k^2}{N_g^2})}$
	Inverse Difference (ID)	The local homogeneity of an image. With more uniform gray levels, the denominator will remain low, resulting in a higher overall value.	$\sum_{k=0}^{N_g-1} \frac{p_{x-y}(k)}{1+k}$
	Inverse Difference Normalized (IDN)	The local homogeneity of an image. IDN normalizes the difference between the neighboring intensity values by dividing over the total number of discrete intensity values.	$\sum_{k=0}^{N_g-1} \frac{p_{x-y}(k)}{1+(\frac{k}{N_g})}$

	Inverse Variance		$\sum_{k=0}^{N_g-1} \frac{p_{x-y}(k)}{k^2}$
	Maximum Probability	The most predominant pair of neighboring intensity values.	$\max(p(i,j))$
	Sum Average	The relationship between occurrences of pairs with lower intensity values and occurrences of pairs with higher intensity values.	$\sum_{k=2}^{2N_g} p_{x+y}(k)k$
	Sum Variance		$\sum_{k=2}^{2N_g} (k - SA)^2 p_{x+y}(k)$
	Sum Entropy	The distribution of neighboring intensity level pairs about the mean intensity level in the GLCM.	$\sum_{i=1}^{N_g}\sum_{j=1}^{N_g} (i - \mu_x)^2 p(i,j)$
Gray Level Dependence Matrix (GLDM) Features (15)	Small Dependence Emphasis	The distribution of small dependencies. A larger value indicates less homogeneous textures.	$\frac{\sum_{i=1}^{N_g}\sum_{j=1}^{N_d} \frac{P(i,j)}{i^2}}{N_Z}$
	Large Dependence Emphasis	The distribution of large dependencies. A larger value means more homogeneous textures.	$\frac{\sum_{i=1}^{N_g}\sum_{j=1}^{N_d} P(i,j)j^2}{N_Z}$
	Gray Level (GL) Non-Uniformity	The similarity of gray-level intensity values in the image	$\frac{\sum_{i=1}^{N_g}\left(\sum_{j=1}^{N_d} P(i,j)\right)^2}{N_Z}$
	Gray Level (GL) Non-Uniformity Normalized		$\frac{\sum_{i=1}^{N_g}\left(\sum_{j=1}^{N_d} P(i,j)\right)^2}{\sum_{i=1}^{N_g}\sum_{j=1}^{N_d} P(i,j)^2}$
	Dependence Non-Uniformity	The similarity of dependence throughout the image.	$\frac{\sum_{j=1}^{N_d}\left(\sum_{i=1}^{N_g} P(i,j)\right)^2}{N_Z}$
	Dependence Non-Uniformity Normalized		$\frac{\sum_{j=1}^{N_d}\left(\sum_{i=1}^{N_g} P(i,j)\right)^2}{{N_Z}^2}$
	Gray Level (GL) Variance	The variance in grey level in the image.	$\sum_{i=1}^{N_g}\sum_{j=1}^{N_d} P(i,j)(i-\mu)^2$ Where $\mu = \sum_{i=1}^{N_g}\sum_{j=1}^{N_d} iP(i,j)$
	Dependence Variance	The variance in dependence size in the image.	$\sum_{i=1}^{N_g}\sum_{j=1}^{N_d} P(i,j)(j-\mu)^2$ Where $\mu = \sum_{i=1}^{N_g}\sum_{j=1}^{N_d} jP(i,j)$
	Dependence Entropy		$\sum_{i=1}^{N_g}\sum_{j=1}^{N_d} p(i,j)\log_2(p(i,j)+\epsilon)$
	Low Gray Level (LGL) Emphasis	The distribution of low gray-level values, with a higher value indicating a greater concentration of low gray-level values in the image.	$\frac{\sum_{i=1}^{N_g}\sum_{j=1}^{N_d} \frac{P(i,j)}{i^2}}{N_Z}$

	High Gray Level (HGL) Emphasis	The distribution of the higher gray-level values, with a higher value indicating a greater concentration of high gray-level values in the image.	$\frac{\sum_{i=1}^{N_g}\sum_{j=1}^{N_d} P(i,j)i^2}{N_Z}$
	Small Dependence Low Gray Level (SDLGL) Emphasis	The joint distribution of small dependence with lower gray-level values.	$\frac{\sum_{i=1}^{N_g}\sum_{j=1}^{N_d} \frac{P(i,j)}{i^2j^2}}{N_Z}$
	Small Dependence High Gray Level (SDGHL) Emphasis	The joint distribution of small dependence with higher gray-level values.	
	Large Dependence Low Gray Level (LDLGL) Emphasis	The joint distribution of large dependence with lower gray-level values.	$\frac{\sum_{i=1}^{N_g}\sum_{j=1}^{N_d} \frac{P(i,j)j^2}{i^2}}{N_Z}$
	Large Dependence High Gray Level (LDHGL) Emphasis	The joint distribution of large dependence with higher gray-level values.	$\frac{\sum_{i=1}^{N_g}\sum_{j=1}^{N_d} P(i,j)i^2j^2}{N_Z}$
Neighboring Gray Tone Difference Matrix (NGTDM) Features (5)	Coarseness	An indicator of the spatial rate of change. Higher value indicates lower spatial change rate and a locally more uniform texture.	$\frac{1}{\sum_{i=1}^{N_g} p_i s_i}$
	Contrast	The spatial intensity change depending on the overall gray level dynamic range.	$(\frac{1}{N_{g,p}(N_{g,p}-1)}\sum_{i=1}^{N_g}\sum_{j=1}^{N_g} p_i p_j (i-j)^2) \times (\frac{1}{N_{v,p}}\sum_{i=1}^{N_g} S_i)$
	Busyness	The change from a pixel to its neighbor. A high value indicates a rapid changing.	$\frac{\sum_{i=1}^{N_g} p_i s_i}{\sum_{i=1}^{N_g}\sum_{j}^{N_g} \lvert ip_i - jp_j \rvert}$
	Complexity	The non-uniformity and busyness of the image.	$\frac{1}{N_{v,p}}\sum_{i=1}^{N_g}\sum_{j}^{N_g} \lvert i-j \rvert \frac{p_i s_i + p_j s_j}{p_i + p_j}$
	Strength	A greater value means slow change in intensity but larger coarse differences in gray level intensities.	$\frac{\sum_{i=1}^{N_g}\sum_{j}^{N_g}(p_i + p_j)(i-j)^2}{\sum_{i=1}^{N_g} s_i}$

Supplementary Table 2. The three-fold cross-validation performance of single networks. It should be noted that the data were obtained by combining the training, testing, and validation set from the Med-Seg (medical segmentation) COVID-19 dataset, and then splitting the combined data into 3 folds.

Three-fold Cross-Validation Performance for Single Segmentation						
	Single U-Net		Single SInfNet		Single SSInfNet	
	Mean	Error	Mean	Error	Mean	error
F1	0.39	0.05	**0.76**	0.04	0.72	0.04
IoU	0.28	0.04	**0.64**	0.04	0.60	0.04
Recall	0.38	0.05	0.77	0.04	0.77	0.04
Precision	0.41	0.05	**0.79**	0.04	0.76	0.02

Supplementary Table 3. The three-fold cross validation performance of multi networks

	**Cross-Validation Performance for Multi Segmentation**						
		Multi-UNet		Multi-SInfNet		Multi-SSInfNet	
		Mean	Error	Mean	error	Mean	Error
GGO	F1	0.26	0.04	0.69	0.06	**0.70**	0.06
	IoU	0.17	0.03	0.63	0.06	**0.64**	0.06
	Recall	0.25	0.03	**0.77**	0.05	0.72	0.06
	Precision	0.3	0.04	0.73	0.05	**0.79**	0.05
Consolidation	F1	0.18	0.04	0.39	0.07	**0.61**	0.07
	IoU	0.13	0.03	0.33	0.06	**0.55**	0.07
	Recall	0.21	0.04	0.45	0.06	**0.68**	0.07
	Precision	0.19	0.04	**0.82**	0.04	0.74	0.07
Background	F1	1	0	1	0	1	0
	IoU	1	0	1	0	1	0
	Recall	1	0	1	0	1	0
	Precision	1	0	1	0	1	0
Overall	F1	0.48	0.03	0.69	0.04	**0.77**	0.04
	IoU	0.43	0.02	0.65	0.04	**0.73**	0.04
	Recall	0.49	0.02	0.74	0.04	**0.80**	0.04
	Precision	0.5	0.03	**0.85**	0.03	0.84	0.04

.

Supplementary Table 4. Comparison with transfer learning based FCN8 network. Quantitative result of Ground-glass Opacities & Consolidation on the test data set of the Med-Seg (medical segmentation) COVID-19 dataset. Prior was obtained from the single segmentation InfNet.

Methods		Multi FCN8		Multi U-Net		Multi SInfNet		Multi SSInfNet	
		mean	error	mean	error	mean	error	mean	error
GGO	F1	0.41	0.059	0.26	0.057	0.38	0.054	**0.43**	0.057
	IoU	0.3	0.046	0.18	0.043	0.27	0.042	**0.31**	0.046
	Recall	0.45	0.066	0.216	0.053	**0.58**	0.065	**0.58**	0.072
	Precision	0.52	0.06	0.405	0.085	0.41	0.058	**0.48**	0.059
Cons	F1	0.42	0.092	0.35	0.097	0.29	0.078	**0.46**	0.096
	IoU	0.33	0.082	0.26	0.08	0.22	0.068	**0.36**	0.088
	Recall	0.56	0.097	0.32	0.089	**0.61**	0.099	0.56	0.11
	Precision	0.51	0.103	0.46	0.116	0.31	0.084	**0.56**	0.101
Background	F1	1.0	0.002	0.857	0.01	1.0	0.002	**1.00**	0.002
	IoU	0.99	0.003	0.754	0.017	0.99	0.003	**0.99**	0.003
	Recall	1.0	0.001	0.998	0.001	0.99	0.002	**0.99**	0.002
	Precision	0.99	0.002	0.755	0.017	1.0	0.002	**1.00**	0.002
Overall	F1	0.61	0.051	0.49	0.055	0.55	0.044	**0.63**	0.052
	IoU	0.54	0.044	0.40	0.046	0.5	0.038	**0.55**	0.046
	Recall	0.67	0.055	0.51	0.048	**0.73**	0.055	0.71	0.061
	Precision	0.67	0.055	0.54	0.073	0.57	0.048	**0.68**	0.054

Supplementary Table 5. Model performance on independent COVID-19 CT Dataset set 2

A: Single InfNet

	SInfNet		SSInfNet	
	Mean	Error	Mean	error
F1	**0.8**	**0.011**	0.78	0.028
IoU	**0.67**	**0.016**	0.64	0.038
Recall	0.79	**0.014**	**0.84**	0.017
Precision	**0.82**	**0.038**	0.73	0.061

B: Multi InfNet

		Multi-SInfNet		Multi-SSInfNet	
		Mean	Error	Mean	error
GGO	F1	**0.79**	**0.056**	0.70	0.066
	IoU	**0.72**	**0.064**	0.61	0.065
	Recall	**0.77**	**0.064**	0.67	0.07
	Precision	0.89	0.043	**0.89**	**0.038**
Consolidation	F1	**0.48**	0.101	0.38	**0.096**
	IoU	**0.39**	0.092	0.32	**0.085**
	Recall	0.70	0.104	**0.78**	**0.096**
	Precision	**0.52**	0.107	0.38	**0.097**
Background	F1	1	0	1	0
	IoU	1	0	1	0
	Recall	1	0	1	0
	Precision	1	0	1	0
Overall	F1	**0.76**	**0.052**	0.70	0.054
	IoU	**0.70**	0.052	0.64	**0.05**
	Recall	**0.82**	0.056	0.81	**0.055**
	Precision	**0.80**	0.05	0.76	**0.045**

Supplementary Table 6. Model performance on the independent COVID-19 CT Data set 3

A: Single InfNet

	SInfNet		SSInfNet	
	Mean	Error	Mean	error
F1	**0.96**	0.002	0.58	0.009
IoU	**0.93**	0.003	0.41	0.009
Recall	**0.96**	0.001	0.53	0.007
Precision	**0.97**	0.005	0.64	0.013

B: Multi InfNet

		Multi-SInfNet		Multi-SSInfNet	
		Mean	Error	Mean	error
GGO	F1	0.94	0.019	**0.94**	0.017
	IoU	0.89	0.029	**0.90**	0.028
	Recall	0.94	0.022	**0.99**	0.002
	Precision	**0.94**	0.019	0.91	0.028
Consolidation	F1	0.11	0.05	**0.13**	0.06
	IoU	0.07	0.037	**0.09**	0.044
	Recall	0.10	0.046	**0.10**	0.048
	Precision	0.20	0.079	**0.73**	0.114
Background	F1	0.95	0.011	**0.98**	0.001
	IoU	0.91	0.019	**0.97**	0.002
	Recall	0.98	0.002	**0.98**	0.002
	Precision	0.93	0.02	**0.99**	0.001
Overall	F1	0.67	0.027	**0.69**	0.026
	IoU	0.62	0.029	**0.65**	0.024
	Recall	0.68	0.024	**0.69**	0.017
	Precision	0.69	0.039	**0.87**	0.048

Supplementary Table 7. Results of ablation studies. The performance of the ablation of our proposed multi-SSInfNet. Multi-SSInfNet refers to the self-supervised SInfNet with Focal Loss and Lookahead optimizer. We tried a variety of the model with a subtraction of the different technologies to carry out the ablation.

		Multi-SInfNet		Multi-SSInfNet – Focal - Lookahead		Multi-SSInfNet - lookahead		Multi-SSInfNet - Focal		Multi-SSInfNet	
		Mean	Error	Mean	Error	Mean	Error	Mean	error	Mean	Error
GGO	F1	0.38	0.054	0.39	0.057	0.36	0.055	0.36	0.056	**0.43**	0.057
	IoU	0.27	0.062	0.29	0.045	0.26	0.044	0.26	0.044	**0.31**	0.046
	Recall	0.58	0.045	0.59	0.071	**0.60**	0.066	0.58	0.071	0.58	0.072
	Precision	0.41	0.058	0.44	0.06	0.38	0.058	0.39	0.058	**0.48**	0.059
Consolidation	F1	0.29	0.078	**0.47**	0.1	0.39	0.091	0.42	0.093	0.46	0.096
	IoU	0.22	0.068	**0.37**	0.093	0.32	0.082	0.32	0.083	0.36	0.088
	Recall	**0.61**	0.099	0.54	0.118	0.52	0.112	0.59	0.106	0.56	0.11
	Precision	0.31	0.084	**0.61**	0.104	0.51	0.104	0.49	0.102	0.56	0.101
Background	F1	1	0.002	1	0.002	1	0.002	1	0.002	**1**	0.002
	IoU	0.99	0.003	0.99	0.003	0.99	0.003	0.99	0.003	**0.99**	0.003
	Recall	0.99	0.002	0.99	0.002	0.99	0.002	0.99	0.002	**0.99**	0.002
	Precision	1	0.002	1	0.002	1	0.002	1	0.002	**1**	0.002
Overall	F1	0.55	0.044	0.62	0.053	0.58	0.049	0.59	0.068	**0.63**	0.052
	IoU	0.50	0.038	0.55	0.047	0.52	0.043	0.52	0.049	**0.55**	0.046
	Recall	**0.73**	0.055	0.71	0.064	0.70	0.06	0.72	0.075	0.71	0.061
	Precision	0.57	0.048	0.68	0.055	0.63	0.054	0.63	0.075	**0.68**	0.054

Supplementary Table 8. Computational costs of processing one image

	Computational cost (seconds) of different methods		
Epoch	FCN8	Multi-SInfNet	Multi-SSInfNet
1	36.61s	49.68	50.28
2	35.73	51.03	50.41
3	36.03	50.38	50.17
4	36.69	48.52	50.34
5	38.51	48.08	52.19
6	37.84	48.26	52.88
7	35.91	48.47	51.88
8	36.01	49.38	53.73
9	35.63	49.69	54.26
10	36.11	48.65	54.43
Average	36.051	49.21	52.06
Relative*	0.742	1	1.06

* The computation analysis was calculated relatively to the baseline multi SInfNet.
